# Multimodal EEG and Eye-Tracking Assessment of Perceived Mental Workload and Situation Awareness in a Visual Display Terminal Simulation Task

**DOI:** 10.3390/s26154835

**Published:** 2026-07-31

**Authors:** Mingchong Xiao, Zhengwen Zhou, Han Peng, Zhe Lin, Yibo Yang, Jialong Chen, Liangjie Guo

**Affiliations:** 1 School of Automation Science and Engineering, South China University of Technology, Guangzhou 510641, China; 202330461721@mail.scut.edu.cn; 2 Faculty of Engineering, China University of Geosciences (Wuhan), Wuhan 430074, China; 1202420512@cug.edu.cn (Z.L.); y15939825816@163.com (Y.Y.); 15671551163@163.com (J.C.); 3 Yangtze Optical Fiber and Cable Joint Stock Limited Company, Wuhan 430073, China; penghan@yofc.com

**Keywords:** visual display terminal, situation awareness, perceived mental workload, EEG, eye-tracking, neuroergonomics

## Abstract

Visual display terminal (VDT) tasks are common in safety-critical systems, where operators must continuously monitor dynamic information and maintain situation awareness (SA). This study examined whether EEG and eye-tracking technologies could support the assessment of SA and perceived mental workload in VDT tasks. Short-term incentive conditions were included only as an exploratory contextual factor. Thirty participants completed a SATEST-based VDT simulation task while subjective scales, behavioral performance, EEG signals, and eye-tracking data were collected during the perception, comprehension, and projection stages of SA. Physiological indicators associated with SA differences and workload-related patterns were examined using SART-based and NASA-TLX-based grouping, supplemented by continuous-score Spearman correlation and exploratory linear regression analyses. The incentive analysis was exploratory, underpowered for small-to-medium effects, and was not designed to validate an incentive-based cognitive-state regulation strategy. Saccade-related eye-tracking indicators differed between relatively higher-SA and lower-SA groups across several stages (*p* = 0.0043 to 0.0457, Cohen’s *d* = −0.670 to −1.002), while fronto-central relative theta power showed an SA-related difference during the SA1 perception stage (*p* = 0.0315, Cohen’s *d* = −0.746). Relatively higher perceived workload was associated with lower SART scores (*p* = 0.0187), lower SA2 accuracy (*p* = 0.0175), larger SA3 angular error (*p* = 0.0151), and lower frontal theta-band and alpha-band absolute power across selected stages (*p* = 0.0094 to 0.0294). The exploratory incentive analysis showed only a limited stage-specific difference in SA1 distance error, with no reliable differences in overall SART score, SA2 accuracy, SA3 angular error, or NASA-TLX score. Thus, the findings provide insufficient evidence that the present short-term incentive manipulation produced reliable cognitive-state regulation. Overall, these findings provide preliminary support for multimodal physiological assessment in VDT tasks and suggest that the identified physiological measures should be regarded as candidate indicators requiring further validation.

## 1. Introduction

### 1.1. VDT Tasks, Operator Safety, and Situation Awareness

With the rapid development of information technology, digital interfaces, and automation, visual display terminal (VDT) tasks have become a common mode of human–computer interaction in modern monitoring and control work [1,2,3]. In safety-critical and safety-relevant systems, such as air traffic management [4], nuclear power plant operation [1], process control [5], transportation monitoring [6], intelligent mining [2], medical supervision [7], and emergency dispatching [8], operators are required to continuously monitor dynamic information, detect abnormal events, and make timely decisions through screen-based interfaces. Although automation can reduce certain physical demands, it often shifts operators’ work from direct manual control to supervisory monitoring, information integration, and cognitive decision-making [9,10]. Therefore, the performance of VDT-based work depends not only on system automation and interface design, but also on the operator’s ability to maintain an accurate understanding of the evolving task environment.

Situation awareness (SA) is widely regarded as a core cognitive prerequisite for effective performance in dynamic human–machine systems. According to Endsley’s three-level model [11,12], SA refers to the perception of relevant elements in the environment, the comprehension of their meaning, and the projection of their future status. This model is particularly applicable to VDT tasks because operators must continuously identify critical visual elements, understand their task-related implications, and anticipate possible future changes. In monitoring work, insufficient SA may lead to missed abnormal cues, delayed responses, misinterpretation of system states, or incorrect predictions of future developments. Previous studies [1,2] have shown that SA degradation is associated with reduced task performance and increased human error risk. Thus, maintaining SA is essential for supporting decision-making and reducing operational errors in VDT-based tasks. However, VDT tasks impose substantial cognitive and visual demands on operators. They often involve prolonged screen viewing, continuous attention allocation, rapid information updating, and simultaneous processing of multiple visual cues. Under such conditions, operators may experience increased mental workload, visual fatigue, and reduced attentional stability. According to multiple resource theory [13], human information-processing capacity is limited, and excessive task demands can create competition for perceptual, cognitive, and response resources. When perceived workload increases, operators may need to invest additional cognitive effort to maintain task performance. However, this compensatory effort may reduce the cognitive resources available for SA, especially for higher-level comprehension and projection processes. Workload-related cognitive burden may increase the probability of delayed responses, incorrect judgments, and errors [14]. In this study, perceived mental workload and SA were therefore treated as related but distinct constructs: perceived workload reflected the operator’s subjective cognitive burden, whereas SA referred to the perception, comprehension, and projection of task-relevant information.

Recent studies have provided empirical evidence for the relationship between VDT-related fatigue, mental workload, and SA degradation [15]. Guo et al. [1] reported that VDT fatigue in nuclear power plant control-room tasks reduced operators’ perception and comprehension abilities, leading to a decline in SA. Sun et al. [2] found that brain fatigue among intelligent coal mine VDT operators impaired sustained attention, suggesting that prolonged VDT operation may weaken continuous monitoring performance. In air traffic control tasks, Li et al. [16] demonstrated that SA-related neurophysiological patterns under different workload conditions could be characterized and SA loss could be recognized using combined electroencephalography (EEG) and eye-tracking features. Similarly, Aricò et al. [17] demonstrated the feasibility of an EEG-based passive brain–computer interface for assessing mental workload in professional air traffic controllers during realistic operational tasks. Kästle et al. [18] further reported associations between SA and EEG signals, supporting the use of neurophysiological measures as complementary indicators of operator cognitive state. Despite these advances, the assessment of SA and workload in VDT tasks still relies on subjective scales and task performance indicators. Although these methods are valuable, they provide limited information about the cognitive processes underlying performance. Subjective ratings are usually retrospective and may be affected by memory bias, self-evaluation bias, and individual differences, whereas task performance mainly reflects final behavioral outcomes and may fail to reveal hidden cognitive effort before performance deterioration occurs [19]. Therefore, objective and process-oriented methods are needed to complement traditional assessment approaches. Multimodal physiological sensing, especially the combined use of EEG and eye-tracking, provides a promising approach for capturing operators’ neural activity, visual attention, workload-related changes, and SA-related differences during VDT tasks [20,21].

Based on this background, the present study focuses on VDT tasks and investigates the assessment of SA and perceived mental workload using a multimodal measurement framework. By integrating subjective scales, behavioral performance, EEG, and eye-tracking indicators, this study aims to identify candidate physiological indicators associated with SA differences and to examine how perceived workload is related to SA during dynamic VDT task performance. Short-term incentive conditions were included only as an exploratory contextual factor to examine whether reward and punishment instructions were associated with SA and perceived workload under the present task conditions. This exploratory analysis was not designed or powered to validate an incentive-based cognitive-state regulation strategy. The findings are expected to provide preliminary evidence for technology-assisted cognitive-state assessment and to inform future research on operator-state assessment and workload-support approaches.

### 1.2. Limitations of Subjective and Performance-Based SA Assessment

SA and mental workload in VDT tasks have traditionally been assessed using subjective scales and task performance measures. Subjective assessment methods, such as the Situation Awareness Rating Technique (SART) [22], the Situation Awareness Global Assessment Technique (SAGAT) [23], the Situation Present Assessment Method (SPAM) [24], and the NASA Task Load Index (NASA-TLX) [25], have been used because they are easy to implement and can provide direct information about operators’ perceived cognitive state [26]. In particular, SART allows participants to retrospectively evaluate their SA after task completion, while NASA-TLX provides a multidimensional evaluation of perceived workload, including mental demand, temporal demand, effort, frustration, and perceived performance. These methods have contributed substantially to the understanding of SA and workload in aviation, transportation, process control, and other human–machine interaction domains.

However, subjective methods have several inherent limitations when applied to VDT-based monitoring tasks. First, most subjective scales are administered after task completion and therefore cannot capture moment-to-moment changes in operators’ cognitive states during task execution. This limitation is particularly relevant in dynamic VDT tasks, where workload and SA may fluctuate with information density, task difficulty, time pressure, and abnormal events. Second, subjective ratings may be influenced by memory bias, self-evaluation bias, individual response tendencies, and differences in scale interpretation. Operators may underreport workload because of overconfidence or professional experience, or overreport workload because of stress, anxiety, or unfamiliarity with the task [27]. Consequently, subjective scores may not fully represent the dynamic cognitive processes supporting perception, comprehension, and projection.

Task performance indicators, such as accuracy, error rate, missed alarms, and tracking deviation, provide more objective behavioral evidence than subjective scales. In VDT tasks, these measures can reflect whether operators correctly detect visual information, understand task-relevant cues, and predict future states. Therefore, performance-based assessment is often used as an indirect indicator of SA. Nevertheless, performance measures primarily reflect task outcomes rather than directly measuring the operator’s internal representation of the situation. Performance can also be influenced by experience, task strategy, motor execution, fatigue, motivation, interface familiarity, and other factors [28,29]. Consequently, acceptable performance may occur despite inadequate SA, whereas poor performance does not necessarily indicate poor SA. Operators may also maintain acceptable accuracy by investing greater cognitive effort, narrowing attention, or adopting compensatory strategies, even when perceived workload has increased. In such cases, observable performance may remain stable while cognitive reserve is reduced. This performance–workload dissociation is particularly important in safety-critical and safety-relevant VDT tasks because visible performance deterioration may emerge only after cognitive demands have accumulated. These ambiguities make it difficult to determine whether an observed error originates from insufficient perception of relevant information, incorrect comprehension of its meaning, failure to project future states, increased workload, or other task-related factors [30,31]. For VDT tasks based on continuous monitoring and rapid information updating, such diagnostic limitations restrict the effectiveness of subjective and behavioral assessment methods [28]. In addition, SA measurement techniques may interfere with the primary task. For example, SAGAT requires task freezing and query-based probing, while SPAM introduces real-time questions during task execution. Although these methods can provide information about operators’ knowledge of the task environment, they may interrupt the natural flow of VDT monitoring, alter attention allocation, and increase additional cognitive load [32]. This issue is relevant for VDT-based monitoring and control tasks, where the assessment method itself should not disturb the operator or change the task context. Therefore, although subjective and performance-based methods remain valuable components of experimental assessment, they are limited in their ability to provide unobtrusive and continuous assessment of SA during VDT task execution.

These limitations have encouraged the use of physiological technologies to complement traditional SA and workload assessment [33]. Physiological signals may provide time-sensitive and process-oriented information about operators’ neural activity, visual attention, and cognitive effort during task execution. Among these technologies, EEG and eye-tracking are suitable for VDT tasks. EEG captures neural activity associated with attention, workload, fatigue, and cognitive control, while eye-tracking characterizes visual search patterns, fixation behavior, saccadic activity, and pupil responses during screen-based information processing. Compared with subjective scales and task performance measures alone, EEG and eye-tracking may provide complementary evidence of cognitive and visual information-processing states before or alongside observable performance changes. Therefore, a multimodal framework integrating subjective scales, behavioral performance, EEG, and eye-tracking may provide a more comprehensive assessment of SA and perceived mental workload. Subjective scales can provide operators’ perceived workload and self-reported SA, behavioral measures can reflect task outcomes, and physiological technologies can capture neural and visual processes during task execution. Such a multimodal approach is important for VDT tasks, where operators may maintain task performance while experiencing increased workload or reduced cognitive reserve. Based on this rationale, the present study adopts a multimodal measurement framework to assess SA and perceived mental workload in VDT tasks.

### 1.3. EEG and Eye-Tracking Technologies for Cognitive State Assessment

Physiological technologies provide a process-oriented approach to complement subjective and performance-based assessment by capturing operators’ cognitive and attentional states during task execution [34]. In the broader field of workload estimation, recent reviews have highlighted the increasing use of physiological sensors to complement subjective and performance-based workload measures, while also noting challenges related to feature selection, standardization, and generalization across tasks and operators [35]. EEG and eye-tracking are relevant to VDT tasks because they correspond to two key aspects of screen-based work: neural information processing and visual attention allocation [36,37]. EEG records task-related brain electrical activity with high temporal resolution, whereas eye-tracking captures the visual behavior through which operators search, select, and process information from display interfaces. Therefore, combining EEG and eye-tracking may provide complementary evidence for assessing SA and perceived mental workload in VDT tasks. A recent systematic review of concurrent EEG and eye-tracking studies also suggested that these two modalities provide complementary information for monitoring cognitive and physiological states such as workload, fatigue, stress, vigilance, and drowsiness [38].

EEG has been widely used in neuroergonomics, human–computer interaction, and process control to evaluate cognitive workload, fatigue, attention, and task engagement [39,40]. Because EEG signals are related to cortical activity, they can provide information about internal cognitive processing that may not be fully reflected in task performance. In workload-related and attention-related studies, frequency-domain EEG features are extracted from canonical bands, including delta, theta, alpha, beta, and gamma rhythms. Frontal and fronto-central theta activity is often associated with working memory demand, executive control, and sustained attention, whereas alpha activity is closely related to attentional allocation, cortical inhibition, and fatigue-related changes [41,42]. For VDT tasks, these EEG features are valuable because operators must continuously perceive, understand, and predict changes in dynamic visual information, corresponding to the perception, comprehension, and projection stages of SA. Thus, EEG can provide complementary physiological evidence for workload-related neural changes and SA-related cognitive processing.

Eye-tracking provides another important source of information for assessing visual behavior in VDT tasks [43,44]. Since most information in VDT tasks is acquired through visual channels, eye movement patterns can directly reflect how operators allocate attention, search for relevant cues, and process screen-based information. Common eye-tracking indicators include fixation duration, fixation count, saccade amplitude, saccade velocity, blink behavior, and pupil diameter [45]. Fixation-related indicators are generally associated with information processing depth and visual attention allocation, while pupil diameter has often been used as an indicator of cognitive effort, although it is sensitive to illumination and should be interpreted carefully in controlled experimental settings [46]. Among these indicators, saccade-related features are especially relevant for dynamic VDT tasks, where operators must frequently shift gaze among moving objects, task-relevant locations, and response options. Saccade peak velocity and its variability can reflect visual scanning stability and information acquisition efficiency [47]. Unstable or excessive saccadic behavior may indicate less efficient visual search or difficulty in maintaining attention to task-relevant information. Therefore, saccade peak velocity and the standard deviation of saccade peak velocity were selected in this study as key eye-tracking indicators for evaluating SA-related visual behavior [48].

Although EEG and eye-tracking can each provide useful information, either modality alone has limitations. EEG can capture changes in neural activity related to attention, workload, and cognitive control, but it is sensitive to artifacts caused by eye movements, muscle activity, and electrode instability, and it provides limited information about visual search strategies. Eye-tracking can reveal where and how operators visually search for information, but it cannot directly explain the neural processing or cognitive effort underlying that visual behavior. Therefore, EEG and eye-tracking should be regarded as complementary rather than interchangeable modalities. By combining these physiological signals with subjective scales and behavioral performance, multimodal assessment can better determine whether SA differences are reflected in subjective experience, task outcomes, neural processing, visual attention, or a combination of these dimensions [49,50].

To avoid conflating different cognitive constructs, the literature reviewed in this study was interpreted according to the specific construct addressed by each study. Studies on mental workload, fatigue, and cognitive effort were used to support the workload-related background and interpretation, whereas studies on situation awareness were used to support the SA framework and the interpretation of perception, comprehension, and projection processes. Studies combining EEG and eye-tracking were used primarily to support the feasibility of multimodal physiological assessment. Despite these advances, important gaps remain in VDT cognitive-state assessment. Existing studies have examined mental workload [51] and SA separately [52], and many rely on single-modal physiological indicators rather than integrating neural, visual, subjective, and behavioral evidence. Moreover, short-term factors, such as reward and punishment instructions, have rarely been considered as auxiliary conditions in VDT assessment. These gaps motivate this study, which adopts an EEG–eye-tracking-based multimodal framework to assess SA and perceived workload in VDT tasks, while treating incentive conditions as an exploratory factor [53,54].

### 1.4. Objectives and Contributions of This Research

Building on the above gaps, this study employed a SATEST-based VDT simulation task and a multimodal measurement framework integrating subjective scales, behavioral performance, EEG, and eye-tracking technologies. The aim was to assess SA and perceived mental workload in VDT tasks and to examine whether physiological indicators can complement traditional subjective and behavioral measures.

First, this study aimed to examine whether EEG and eye-tracking indicators differed between participants with relatively higher and lower self-reported SA in VDT tasks. By combining group-based descriptive comparisons with continuous-score association analyses, the study sought to identify candidate physiological indicators associated with SA differences. Second, this study examined the relationship between perceived mental workload and SA-related outcomes across subjective, behavioral, EEG, and eye-tracking measures. Because NASA-TLX was administered after task completion and workload was not experimentally manipulated, these analyses were intended to characterize associations. Third, short-term incentive conditions were included as an exploratory contextual factor to determine whether reward and punishment instructions were associated with differences in SA and perceived workload. This analysis was underpowered for small-to-medium effects and was not designed to validate an incentive-based cognitive-state regulation strategy.

**Figure 1:**
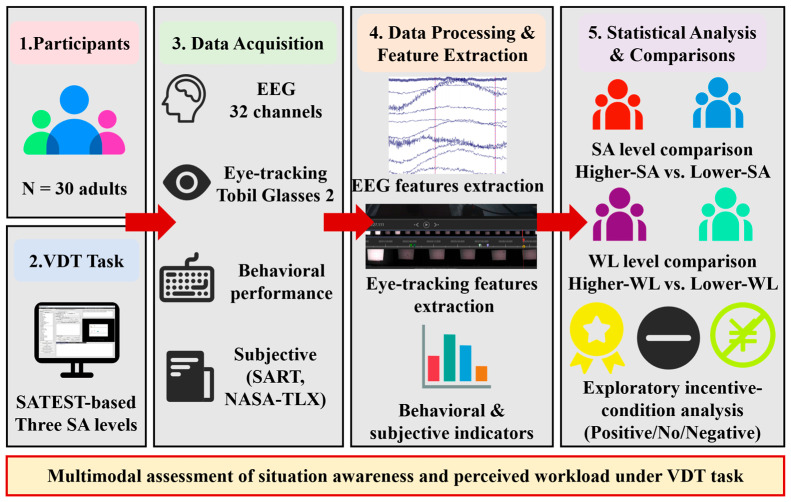
Overall workflow of the multimodal VDT cognitive-state assessment framework.

The main contribution of this study lies in providing preliminary multimodal evidence on the assessment of SA and perceived mental workload in a controlled VDT simulation task. As shown in Figure 1, by integrating EEG and eye-tracking features with traditional SA and workload measures, this study provides a multimodal approach for jointly characterizing observable task outcomes and process-level physiological correlates of cognitive state. In particular, it identifies candidate saccade-related indicators associated with self-reported SA differences and examines workload-related patterns across subjective, behavioral, and frontal EEG measures. The incentive-related findings are retained as exploratory and stage-specific observations. Overall, the proposed measures should be regarded as candidate indicators requiring further validation in larger samples, controlled workload manipulations, and high-fidelity or field-based VDT environments.

## 2. Materials and Methods

### 2.1. Participants

Thirty healthy adults were recruited to participate in the experiment. The participants were 21 ± 3 years old and had normal or corrected-to-normal vision. Before the experiment, all participants were screened according to the following exclusion criteria: use of psychotropic medication, including stimulants, antidepressants, or anxiolytics; history of neurological injury or disease; history of epilepsy or psychiatric disorders; and insufficient sleep before the experiment. To reduce the influence of physiological fluctuations on EEG and eye-tracking signals, participants were required to sleep for at least 8 h before the experiment and to avoid caffeine, alcohol, tea, and tobacco prior to testing. Before the formal experiment, the experimental procedure, task requirements, and data collection methods were explained to each participant. All participants voluntarily took part in the study and provided written informed consent. During the experiment, relevant safety and laboratory procedures were strictly followed. This study was approved by the Ethics Committee of China University of Geosciences (Wuhan) and conducted in accordance with the Declaration of Helsinki. Written informed consent was obtained from all participants prior to the experiment.

### 2.2. Experimental Task: SATEST-Based VDT Simulation

A VDT simulation experiment was designed based on the Situation Awareness Test (SATEST) implemented in PEBL version 2.1 [55]. SATEST is a dynamic visual monitoring and tracking task that can be used to assess participants’ ability to perceive, understand, and predict the state of moving visual objects [56]. It is suitable for simulating VDT operations because both SATEST and typical VDT tasks require participants to continuously monitor screen-based information, detect dynamic visual changes, understand task-relevant elements, and predict future states.

**Figure 2:**
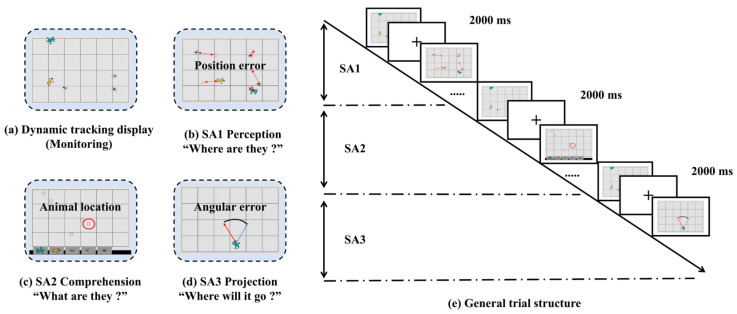
SATEST-based VDT task paradigm corresponding to the three levels of SA. (**a**) Participants monitored five moving objects in a grid. (**b**) The SA1 perception task required participants to indicate the final object locations. (**c**) The SA2 comprehension task required participants to identify object identities at highlighted locations. (**d**) The SA3 projection task required participants to predict the future movement direction of a target object. (**e**) General trial structure of the SATEST-based VDT simulation task. The correct direction of movement is shown in red, whereas the selected direction is shown in blue. The three task phases were used to operationalize Endsley’s three SA levels: perception, comprehension, and projection. The diagonal sequence in panel (**e**) indicates the temporal progression of a trial. Red markings denote task-relevant reference information, blue markings denote the participant’s response, and red circles indicate highlighted target locations.

As shown in Figure 2, SATEST was used as a controlled VDT-like simulation task to measure the three components of situation awareness under dynamic screen-based monitoring conditions. The task requires participants to continuously monitor multiple moving visual objects, retain their spatial locations and identities, and predict future movement directions. These requirements correspond to key cognitive demands in VDT scenarios, such as visual information acquisition, task-relevant cue integration, working-memory maintenance, and projection of future system states.

The three response phases of SATEST were mapped onto Endsley’s three-level SA model. The SA1 perception phase measured whether participants could perceive and localize task-relevant visual elements by asking them to indicate the final positions of the moving objects. The SA2 comprehension phase measured whether participants could understand and retain the meaning of task-relevant elements by associating object identities with previously highlighted locations. The SA3 projection phase measured whether participants could anticipate future states by predicting the future movement direction of a target object. Therefore, SATEST provided a structured and repeatable paradigm for assessing perception, comprehension, and projection within the same VDT-like task environment.

In the task, five insect-like objects moved randomly within a grid displayed on the computer screen. Participants were instructed to continuously monitor the positions, identities, and movement directions of the objects. Following the principle of the Situation Awareness Global Assessment Technique (SAGAT), the moving objects disappeared from the screen after a random period, and participants were required to answer questions related to the most recent state of the objects. These questions were designed to correspond to the three levels of SA: perception, comprehension, and projection.

For the SA1 perception task, after the five objects disappeared, participants were asked to click on the final locations of the objects before disappearance. The system recorded the average distance error between the selected and actual object locations. A larger distance error indicated poorer perceptual SA.

For the SA2 comprehension task, after the objects disappeared, two target locations were sequentially highlighted by red circles. Participants were required to identify the object types that had appeared at the highlighted locations and select the corresponding object identities from the options displayed at the bottom of the screen. The system recorded response accuracy. A lower accuracy indicated poorer comprehension SA.

For the SA3 projection task, after the objects disappeared, participants were asked to predict the future movement direction of a specified object based on its previous movement trajectory. The system recorded the angular error between the predicted direction and the actual direction. A larger angular error indicated poorer projection SA.

Each participant first completed a practice session to become familiar with the task procedure. The practice session included three trials for each task type, and these data were not included in the subsequent analysis. In the formal experiment, each SA task type was repeated 15 times. During the task, EEG and eye-tracking signals were recorded continuously, and task performance data were logged by the software.

### 2.3. Experimental Design and Grouping Strategy

**Figure 3:**
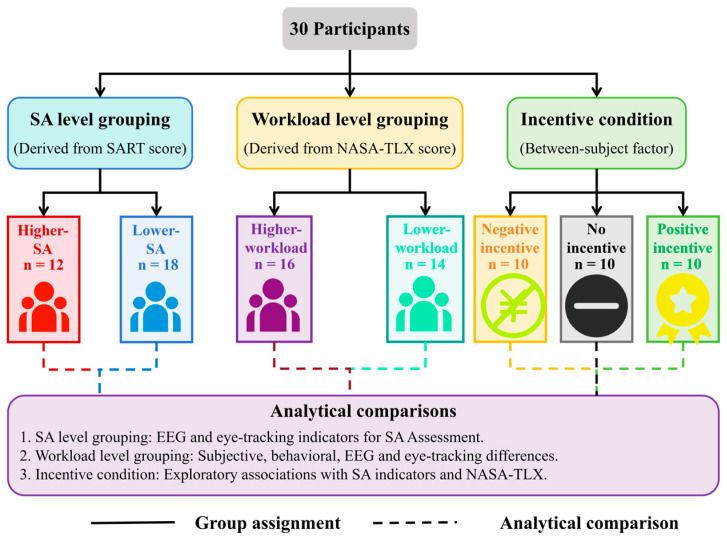
Experimental design and analytical grouping strategy.

Based on the above SATEST-SA mapping, the experimental design treated SA1, SA2, and SA3 as stage-specific behavioral and physiological analysis windows corresponding to perception, comprehension, and projection, respectively. The experiment was designed to assess SA and perceived mental workload in a SATEST-based VDT simulation task using a multimodal measurement framework. All participants completed the same objective task while EEG, eye-tracking, subjective scale, and behavioral performance data were collected. The experimental design included three analytical factors: SA level, perceived workload level, and incentive condition, with incentive condition treated as an exploratory factor, as shown in Figure 3.

Incentive condition was included as an exploratory between-subject contextual factor. The 30 participants were assigned to three groups: positive incentive, negative incentive, and no incentive, with 10 participants in each group. In the positive-incentive condition, participants were informed that better task performance would be associated with reward. In the negative-incentive condition, participants were informed that poorer task performance would be associated with punishment. In the no-incentive condition, participants completed the same task without additional reward or punishment information. The task procedure, experimental environment, and data-acquisition protocol were kept consistent across the three conditions. The incentive manipulation was designed to provide a short-term motivational context rather than a sustained or repeatedly reinforced intervention. Given the subgroup size of 10 participants per condition, the study was not designed or powered to detect small-to-medium incentive-related effects. Accordingly, the incentive-related analysis was treated as exploratory and was intended to identify only preliminary and potentially stage-specific patterns, rather than to validate an incentive-based cognitive-state regulation strategy.

Mental workload was evaluated using the NASA-TLX scale after task completion. For visualization and group-based descriptive comparison, participants were categorized into relatively higher and lower workload groups using the sample mean of NASA-TLX scores as the cut-off. This grouping strategy was used to provide an interpretable comparison of subjective SA, behavioral performance, EEG features, and eye-tracking indicators between participants with relatively higher and lower perceived workload. In this study, the relatively higher-workload group included 16 participants, and the relatively lower-workload group included 14 participants. Because NASA-TLX is originally a continuous measure, this group-based comparison was supplemented by continuous-score Spearman correlation and exploratory linear regression analyses using the original NASA-TLX scores to reduce the potential information loss caused by dichotomization.

SA level was also treated as an analytical grouping variable rather than a manipulated factor. For visualization and group-based physiological indicator comparison, participants were categorized into relatively higher and lower SA groups using the sample mean of SART scores as the cut-off. In this study, the relatively higher-SA group included 12 participants, and the relatively lower-SA group included 18 participants. This asymmetric grouping resulted from the empirical distribution of SART scores and was not intended to define strict categorical higher-SA and lower-SA classes. Instead, the group-based comparison was used to provide an interpretable visualization of whether EEG and eye-tracking indicators differed between participants with relatively higher and lower self-reported SA. Because SART is originally a continuous measure, complementary Spearman correlation and exploratory linear regression analyses were further conducted using the original continuous SART scores to reduce the information loss and arbitrary separation caused by dichotomization.

Therefore, the overall experimental design consisted of three complementary analyses. First, SART-based group comparison was used for descriptive visualization and preliminary identification of candidate EEG and eye-tracking indicators associated with SA differences. Second, NASA-TLX-based group comparison was used to examine descriptive differences in SA-related outcomes between relatively higher and lower perceived workload levels. Third, continuous-score Spearman correlation and exploratory linear regression analyses were used to examine whether the associations remained interpretable when SART and NASA-TLX were treated as continuous variables. The three incentive conditions were compared exploratorily to identify preliminary and potentially stage-specific associations with SA and perceived workload.

### 2.4. Apparatus Configuration, Data Acquisition, and Synchronization

EEG and eye-tracking technologies were used to record participants’ physiological responses during the SATEST-based VDT simulation task. The apparatus configuration was designed to capture two complementary aspects of operator cognitive state: neural activity during task processing and visual behavior during screen-based information acquisition.

#### 2.4.1. Apparatus Configuration and Data Acquisition

As shown in Table 1, EEG data were collected using a 32-channel Neuroscan Grael EEG 2 system (Neuroscan, Abbotsford, Australia). Electrodes were positioned according to the international 10–20 system [57]. EEG signals were recorded at a sampling rate of 1024 Hz using Curry 8 software, which was used for signal acquisition and preliminary data management. During preparation, the EEG cap was positioned to ensure stable electrode contact, and the signal quality was checked before the formal task began.

Eye movement data were collected using Tobii Glasses 2 wearable eye-tracking glasses (Tobii, Danderyd, Sweden). The device contains four eye-tracking cameras and supports wireless connection to the computer. Eye-tracking data were recorded at a sampling rate of 50 Hz, and scene video was recorded at a resolution of 1920 × 1080 pixels at 25 fps. The device tracks gaze using pupil–corneal reflection and binocular dark-pupil tracking technology. It provides real-time monitoring and slippage compensation, which helps reduce the influence of positional shifts during the experiment [58]. Eye-tracking data were recorded using Tobii Pro Glasses Controller and processed using Tobii Pro Lab.

Before the formal task, the eye tracker was calibrated according to the manufacturer’s procedure to ensure gaze recording. Participants then sat in front of the computer screen and performed the VDT simulation task while wearing the EEG cap and eye-tracking glasses. During the task, the EEG system, eye-tracking device, and behavioral task software were operated simultaneously to obtain neural activity, visual behavior, and task performance data during the SA1 perception, SA2 comprehension, and SA3 projection stages.

#### 2.4.2. Synchronization and Event Alignment

The SATEST task software generated trial-level event logs for each participant. These logs included participant ID, trial number, SA stage, task-event information, behavioral response, and task outcome. The SATEST event logs were used as the temporal reference for offline alignment of EEG, eye-tracking, and behavioral data. During the experiment, EEG recording, eye-tracking recording, scene video recording, and the SATEST task were started in a fixed sequence before each formal session. The beginning of the formal SATEST task was used as the common reference point for subsequent offline alignment. The event timestamps corresponding to the SA1 perception, SA2 comprehension, and SA3 projection task windows were then converted to EEG sample indices according to the EEG sampling rate of 1024 Hz and to eye-tracking sample indices according to the eye-tracking sampling rate of 50 Hz.

For each participant, EEG and eye-tracking features were extracted within the corresponding SA1, SA2, and SA3 task windows. The SA1 window corresponded to the object-location recall period, the SA2 window corresponded to the object-identity comprehension period, and the SA3 window corresponded to the movement-direction prediction period. Trials with missing task logs, severe gaze loss, or rejected EEG epochs were excluded from the corresponding stage-level feature calculation. Finally, valid EEG, eye-tracking, and behavioral features were averaged within each participant and SA stage for subsequent statistical analysis.

Temporal alignment among the EEG system, eye tracker, and behavioral task software was achieved through interface-based timestamp matching. Task-event logs and corresponding timestamps were recorded during the experiment and then matched offline across the multimodal data streams.

### 2.5. Subjective, Behavioral, and Physiological Data Processing

**Figure 4:**
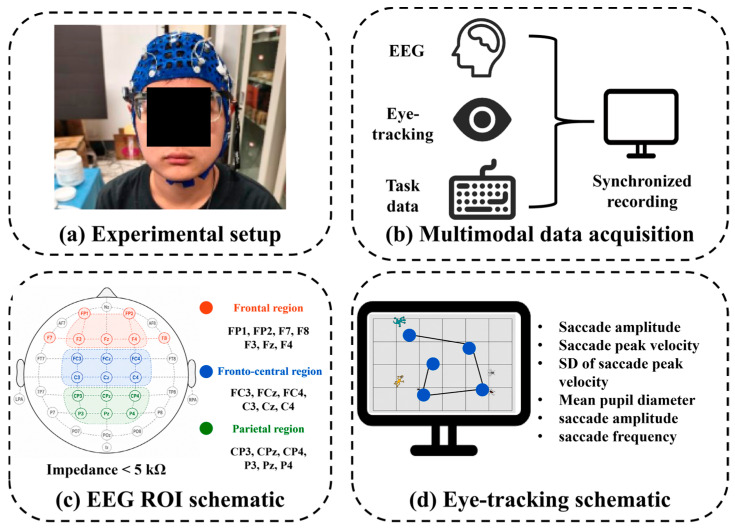
Experimental setup and apparatus configuration. (**a**) Experimental setup. (**b**) Multimodal data acquisition. (**c**) EEG ROI schematic. (**d**) Eye-tracking feature schematic.

Based on the synchronization and event-alignment procedure described above, the collected subjective, behavioral, EEG, and eye-tracking data were organized according to the three SA stages of the SATEST-based VDT task. Subjective scales were used to quantify self-reported SA and perceived workload, behavioral indicators were extracted from the SATEST task software, EEG features were obtained from preprocessed frequency-domain signals, and eye-tracking features were derived from Tobii Pro Lab exported metrics. These data were then organized at the participant level for subsequent statistical analyses, as shown in Figure 4.

#### 2.5.1. Subjective and Behavioral Measures

Subjective SA was measured using the SART. SART is a post-task self-rating method that evaluates operators’ perceived SA from dimensions related to attentional demand, attentional supply, and understanding of the situation. In this study, the SART score was used as the subjective SA indicator.

Subjective mental workload was measured using the NASA-TLX. NASA-TLX evaluates perceived workload across multiple dimensions, including mental demand, physical demand, temporal demand, perceived performance, effort, and frustration. In this study, NASA-TLX scores were used to quantify participants’ perceived workload after the VDT simulation task.

As shown in Table 2, behavioral performance was recorded by the SATEST task software. Three types of behavioral indicators were extracted according to the three SA levels. For SA1 perception, the average distance error between the selected and actual object locations was used as the performance indicator. For SA2 comprehension, the accuracy of identifying the object types at the highlighted locations was used as the performance indicator. For SA3 projection, the angular error between the predicted and actual movement directions was used as the performance indicator. The final behavioral analysis focused on these three SA-specific performance indicators because they directly corresponded to the perception, comprehension, and projection levels of SA.

Subjective scales and behavioral indicators were used together with EEG and eye-tracking features to provide a multimodal assessment of SA and perceived workload. SART and NASA-TLX captured self-reported cognitive states, behavioral indicators reflected task outcomes, and physiological signals provided process-level evidence of neural activity and visual attention during task execution.

#### 2.5.2. EEG Preprocessing and Feature Extraction

EEG data were preprocessed before feature extraction to reduce non-neural noise and artifacts. The preprocessing procedure included visual inspection, ROI-channel selection, re-referencing, filtering, ICA-based artifact correction, event-based epoch segmentation, epoch rejection, baseline correction, 2 s segmentation, and frequency-domain feature extraction [59]. EEG data were processed using MATLAB R2020b and the EEGLAB v2020.0 toolbox [60].

The continuous EEG recordings were first visually inspected to identify channels with persistent abnormal noise, poor contact, or flat-line activity. After preprocessing, EEG features were extracted from predefined regions of interest (ROIs) selected according to the cognitive demands of the SATEST-based VDT task and the hypotheses of the present study. Three ROIs were defined: the frontal region, fronto-central region, and parietal region. The frontal region included FP1, FP2, F7, F8, F3, Fz, and F4; the fronto-central region included FC3, FCz, FC4, C3, Cz, and C4; and the parietal region included CP3, CPz, CP4, P3, Pz, and P4. The frontal and fronto-central regions were selected because they are commonly associated with attentional control, executive processing, working-memory demand, and workload-related theta and alpha activity [61,62]. These functions are relevant to the SATEST task because participants must continuously monitor moving objects, update object-related information, and maintain task goals during perception, comprehension, and projection. The parietal region was included because parietal activity is closely related to visuospatial attention, spatial location maintenance, and visual information processing, which are required for object localization, identity-location association, and movement-direction prediction. Therefore, the selected ROIs were directly linked to the expected cognitive processes underlying SA and perceived workload in the VDT simulation task.

The retained EEG signals were re-referenced using the average reference method. A 0.5 Hz high-pass filter was applied to remove slow baseline drift, a 50 Hz low-pass filter was used to suppress high-frequency noise, and a 48–52 Hz notch filter was applied to reduce power-line interference. Independent component analysis (ICA) [63] was then applied to the continuous EEG data after re-referencing and filtering. ICA was performed in EEGLAB using the infomax-based runica algorithm. Artifact-related independent components were identified by inspection of their scalp topographies, time courses, and power spectra. Components showing frontal-dominant topographies with large transient deflections were identified as eye-blink components. Components showing lateralized frontal distributions and horizontal deflections were identified as horizontal eye-movement components. Components with high-frequency spectral activity and spatially focal or peripheral distributions were identified as muscle-related components. Components with periodic temporal patterns consistent with cardiac activity were identified as cardiac-related components. These artifact-related components were removed, and the cleaned EEG signals were reconstructed from the remaining components. The number of removed components varied across participants depending on recording quality and artifact level.

The cleaned continuous EEG data were segmented according to the SA1 perception, SA2 comprehension, and SA3 projection task windows defined by the SATEST event logs. The event timestamps were converted to EEG sample indices according to the EEG sampling rate of 1024 Hz before epoch extraction. Epochs containing large-amplitude fluctuations, severe movement artifacts, abnormal trends, or residual non-neural artifacts after ICA correction were rejected through inspection. No fixed universal amplitude threshold was applied because signal amplitude varied across participants and electrodes after artifact correction. Instead, epochs were rejected when abnormal waveforms clearly deviated from the surrounding EEG activity or when residual blink, movement, muscle, or electrode artifacts remained visible. Baseline correction was performed by subtracting the mean amplitude of each epoch before spectral analysis to reduce inter-epoch baseline variability. Each valid EEG epoch was divided into 2 s segments for spectral analysis. The 2 s window was selected because it provided 2048 samples at the 1024 Hz sampling rate, corresponding to an approximate frequency resolution of 0.5 Hz. This window length provided sufficient data for stable PSD estimation while maintaining the stage-specific temporal resolution required for the SA1, SA2, and SA3 task periods. Clean EEG segments corresponding to each SA stage were retained for subsequent frequency-domain feature extraction.

Power spectral density (PSD) analysis was used to extract frequency-domain EEG features [64]. PSD was calculated using Bartlett’s averaged periodogram method. For each EEG segment, the signal was divided into shorter sub-segments, the periodogram of each sub-segment was calculated, and the average of these periodograms was used as the final PSD estimate. Compared with a single periodogram, Bartlett’s method reduces the variance of the estimate and provides more stable frequency-domain features.

Frequency-domain EEG features were extracted from four canonical frequency bands: delta (δ: 0.5–4 Hz), theta (θ: 4–8 Hz), alpha (α: 8–13 Hz), and beta (β: 13–30 Hz) [65]. These bands were selected because they provide complementary information about the cognitive and attentional processes involved in the SATEST-based VDT task. Theta-band activity, particularly over frontal and fronto-central regions, has often been associated with executive control, working-memory demand, and sustained attention. Alpha-band activity is related to attentional allocation, cortical inhibition, and fatigue-related changes, whereas beta-band activity may reflect alertness and task engagement. Delta-band activity was retained as part of the canonical low-frequency spectral profile to provide a more complete description of task-related EEG activity.

For each participant, SA stage, and brain region, absolute power, relative power, and ratio-based indices were calculated from the PSD estimates. Absolute power was used to quantify the magnitude of band-specific neural activity, whereas relative power and ratio-based indices were calculated to reduce the influence of inter-individual differences in overall EEG amplitude and to characterize the balance among slow-wave, attentional, and higher-frequency activity. These features were therefore selected to provide complementary evidence for SA-related cognitive processing and perceived workload, rather than to serve as a single diagnostic marker. The absolute power of each frequency band was calculated by summing the PSD values within the corresponding frequency range:

(1) $$AP_{b}=\sum_{f_{i}\in b}PSD(f_{i})\Delta f$$

where $AP_{b}$ denotes the absolute power of frequency band *b*, $PSD(f_{i})$ represents the power spectral density at frequency bin $f_{i}$, and Δf is the frequency resolution. The relative power of each band was then calculated as:

(2) $$RP_{b}=\frac{AP_{b}}{AP_{\delta }+AP_{\theta }+AP_{\alpha }+AP_{\beta }}$$

where $RP_{b}$ denotes the relative power of frequency band *b*. In addition, ratio-based EEG indices were calculated to characterize the balance among different components:

(3) $$R_{\theta /\beta }=\frac{AP_{\theta }}{AP_{\beta }},\;{\;R}_{\alpha /\beta }=\frac{AP_{\alpha }}{AP_{\beta }},\;R_{\left(\theta +\alpha \right)/\beta }=\frac{AP_{\theta }+AP_{\alpha }}{AP_{\beta }}$$

and

(4) $$R_{\left(\theta +\alpha \right)/\left(\beta +\alpha \right)}=\frac{AP_{\theta }+AP_{\alpha }}{AP_{\beta }+AP_{\alpha }}$$

These absolute power, relative power, and ratio-based indices were calculated separately for each participant, SA stage, and brain region. Finally, EEG features were averaged within each brain region and SA stage at the participant level. These features were then used as physiological indicators in the subsequent analyses of SA level, perceived workload level, and incentive condition.

#### 2.5.3. Eye-Tracking Feature Extraction

As shown in Table 3, eye-tracking data were processed to obtain visual behavior indicators during the VDT simulation task. The eye-tracking indicators were derived from Tobii Pro Lab exported metrics to ensure consistency with the device-specific event detection, fixation identification, and saccade classification procedures. The exported eye-tracking metrics were organized according to the same SA1, SA2, and SA3 event windows defined by the SATEST task logs. Five eye-tracking indicators were extracted for each SA stage: mean saccade peak velocity, standard deviation of saccade peak velocity, saccade amplitude, saccade frequency, and pupil diameter [66]. Mean saccade peak velocity was used to describe the average maximum velocity of saccadic eye movements within a given SA stage. The standard deviation of saccade peak velocity was used to describe the variability of saccadic behavior. Saccade amplitude reflected the spatial extent of gaze shifts, and saccade frequency reflected the number of saccades per unit time. Pupil diameter was used as an indicator related to cognitive effort, while recognizing that pupil-based measures may be influenced by illumination and therefore require controlled experimental conditions. These eye-tracking indicators were selected because they reflect different aspects of visual information processing in VDT tasks. Saccade-related indicators describe visual scanning behavior, search efficiency, and attentional stability, while pupil diameter provides complementary information about visual and cognitive effort. In dynamic VDT tasks, unstable or excessive saccadic behavior may indicate inefficient visual search or difficulty maintaining attention to task-relevant information. Therefore, mean saccade peak velocity and the standard deviation of saccade peak velocity were treated as key eye-tracking indicators for SA-related visual behavior.

For each participant, eye-tracking features were averaged separately for the SA1, SA2, and SA3 task periods. These stage-specific indicators were then used together with EEG features, subjective ratings, and performance measures in the statistical analyses.

### 2.6. Statistical Analysis

Statistical analyses were conducted to examine whether EEG and eye-tracking indicators differed between participants with relatively higher and lower self-reported SA, whether perceived workload was associated with SA-related outcomes, and whether incentive conditions were associated with differences in SA and perceived workload. All continuous variables are reported as mean ± standard deviation unless otherwise specified. A significance level of *p* < 0.05 was used for all statistical tests.

First, for visualization and group-based descriptive comparison, participants were categorized into relatively higher-SA and lower-SA groups using the sample mean of SART scores as the cut-off. This grouping was used to provide interpretable group-level visualization rather than to define strict categorical SA classes. Between-group comparisons were conducted for EEG and eye-tracking indicators extracted during the SA1 perception, SA2 comprehension, and SA3 projection stages. Continuous-score Spearman correlation and exploratory linear regression analyses were further conducted using the original SART scores to examine whether the group-based findings were supported when SART was treated as a continuous variable.

Second, for workload-related analyses, participants were categorized into relatively higher-workload and lower-workload groups using the sample mean of NASA-TLX scores as the cut-off. Group-based comparisons were conducted for subjective SA, behavioral SA indicators, EEG features, and eye-tracking indicators. This group-based comparison was supplemented by continuous-score Spearman correlation and exploratory linear regression analyses using the original NASA-TLX scores to reduce the potential information loss caused by dichotomization.

Third, the incentive condition was analyzed as an exploratory between-subject factor with three levels: positive incentive, negative incentive, and no incentive. One-way analysis of variance (ANOVA) was used to compare SART scores, NASA-TLX scores, and behavioral SA indicators among the three incentive groups. When the ANOVA result was significant, post hoc comparisons were conducted to identify differences between specific incentive conditions. Because each incentive group included only 10 participants, incentive-related analyses were treated as exploratory and interpreted cautiously. To clarify the statistical boundary of the current sample size, sensitivity power analyses were conducted. For two-group comparisons with sample sizes similar to the SART-based SA grouping (*n* = 12 and *n* = 18) or NASA-TLX-based workload grouping (*n* = 16 and *n* = 14), the current design had 80% power at α = 0.05 only for large standardized mean differences, approximately Cohen’s *d* = 1.08 and *d* = 1.06, respectively. For the three-group incentive analysis with *n* = 30, the current design was sensitive only to relatively large effects, approximately Cohen’s *f* = 0.60, corresponding to $\eta_{p}^{2}$ = 0.264. These sensitivity results indicate that the present study was not powered to test small-to-medium incentive-related effects. Therefore, findings were interpreted cautiously, and the analyses were treated as preliminary evidence. Non-significant findings were regarded as insufficient evidence for reliable incentive-related cognitive-state regulation, rather than as evidence that incentive conditions were ineffective. Observed differences were treated as preliminary and potentially stage-specific patterns requiring confirmation in a larger and adequately powered study.

To reduce potential information loss caused by dichotomizing continuous SART and NASA-TLX scores, complementary correlation analyses were conducted using the original continuous scores. Spearman rank correlation was used because of the relatively small sample size and the possibility of non-normal distributions in physiological indicators. Specifically, continuous SART scores were correlated with the physiological indicators that showed SA-related group differences. Continuous NASA-TLX scores were correlated with subjective SA, behavioral performance indicators, and EEG indicators that showed workload-related group differences. These analyses were used to examine whether the group-based findings were supported by continuous-score associations. Before group comparisons, data distributions and variance homogeneity were checked. When the assumption of equal variance was violated, Welch’s *t*-test or Welch’s ANOVA was used as appropriate. Effect sizes were reported where applicable. Cohen’s *d* was calculated for two-group comparisons, and partial eta squared ($\eta_{p}^{2}$) was calculated for ANOVA. Physiological results were interpreted based on statistical significance, effect size, and their relevance to SA and workload assessment. Incentive-related analyses were treated as exploratory. Because complete trial-level response-time records were not available in the final exported dataset, response time was not included in the statistical analyses. The behavioral analyses therefore focused on SA1 distance error, SA2 accuracy, and SA3 angular error.

In addition to Spearman correlation analyses, exploratory linear regression analyses were conducted using the original continuous SART and NASA-TLX scores. For SA-related analyses, the continuous SART score was used as the dependent variable, and the significant physiological indicators identified in the group-based comparisons were separately entered as predictors. For workload-related analyses, the continuous NASA-TLX score was used as the dependent variable, and workload-related subjective, behavioral, and EEG indicators were separately entered as predictors. Separate simple regression models were used instead of high-dimensional multiple regression models because of the limited sample size. These regression analyses were used to examine whether the observed associations remained interpretable when SART and NASA-TLX were treated as continuous variables rather than dichotomized groups.

The group-based comparisons were therefore interpreted as descriptive and visualization-oriented analyses, whereas the continuous-score Spearman correlation and exploratory linear regression analyses were used as supplementary evidence to reduce the limitations of dichotomizing SART and NASA-TLX scores.

## 3. Results

The results are presented according to the three analytical objectives of this study. First, EEG and eye-tracking indicators were examined to determine whether physiological features differed between participants with relatively higher and lower self-reported SA. Second, subjective, behavioral, EEG, and eye-tracking measures were compared between relatively higher-workload and lower-workload groups to evaluate the association between perceived mental workload and SA-related outcomes. Third, incentive conditions were analyzed exploratorily to examine whether short-term reward and punishment instructions were associated with differences in SA and perceived workload. For clarity and consistency, all group-based results are reported as mean ± SD in tables, with Welch’s *t*-test statistics and Cohen’s *d* for two-group comparisons. For ANOVA-based incentive analyses, *F* values, *p* values, and partial eta squared ($\eta_{p}^{2}$).

### 3.1. EEG and Eye-Tracking Indicators Associated with SA Differences

To examine SA-related physiological differences, participants were categorized into relatively higher-SA and lower-SA groups according to their SART scores. This group-based comparison was used for descriptive visualization and preliminary indicator screening, and was supplemented by continuous-score correlation and regression analyses. Eye-tracking and EEG features extracted during the SA1 perception, SA2 comprehension, and SA3 projection stages were compared between the two groups. The purpose of this analysis was to identify physiological indicators that were sensitive to SA differences in the VDT task.

**Figure 5:**
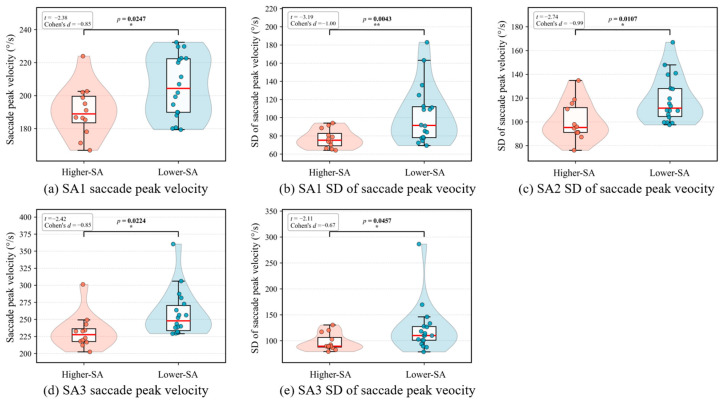
Eye-tracking indicators differing between relatively higher-SA and lower-SA groups. (**a**) SA1 mean saccade peak velocity (°/s), (**b**) SA1 SD of saccade peak velocity (°/s), (**c**) SA2 SD of saccade peak velocity (°/s), (**d**) SA3 saccade peak velocity (°/s), and (**e**) SA3 SD of saccade peak velocity (°/s). Statistical annotations indicate *p* values and Cohen’s *d*. * *p* < 0.05; ** *p* < 0.01.

The eye-tracking results showed that saccade-related indicators were sensitive to SA levels. As shown in Table 4 and Figure 5, the standard deviation of saccade peak velocity differed significantly between the relatively higher-SA and lower-SA groups during the SA1 perception stage (*p* = 0.0043) and the SA2 comprehension stage (*p* = 0.0107). In addition, mean saccade peak velocity showed significant group differences during the SA1 perception stage (*p* = 0.0247) and the SA3 projection stage (*p* = 0.0224). The standard deviation of saccade peak velocity during the SA3 projection stage also reached statistical significance (*p* = 0.0457).

Overall, the lower-SA group showed higher saccade peak velocity and greater variability in saccade peak velocity than the higher-SA group across the significant eye-tracking indicators. These differences showed moderate-to-large effect sizes, with Cohen’s *d* values ranging from −0.670 to −1.002. This pattern suggests that lower SA was associated with less stable visual scanning behavior during the dynamic VDT task. Since the SATEST task required participants to continuously monitor moving objects, retain their spatial positions, identify object information, and predict future movement directions, unstable saccadic behavior may reflect reduced efficiency in visual information acquisition and attention allocation. Therefore, mean saccade peak velocity and the standard deviation of saccade peak velocity showed the clearest SA-related differences among the selected eye-tracking indicators in this dataset.

**Figure 6:**
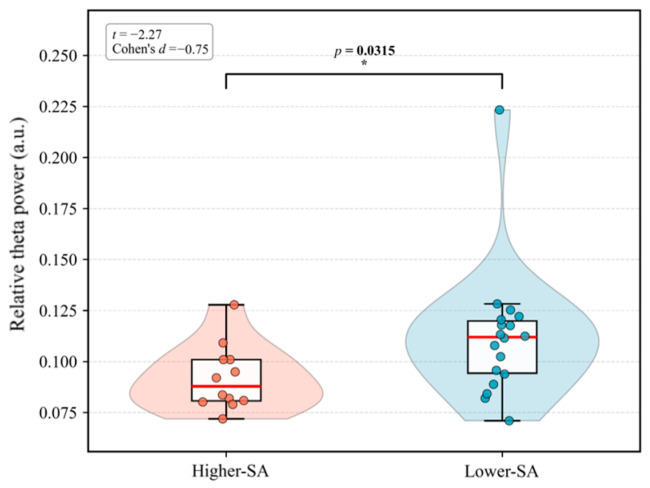
Fronto-central relative theta power (a.u.) during SA1 in relatively higher-SA and lower-SA groups. Group labels were defined using the sample mean of SART scores for visualization. Statistical annotations indicate *p* value and Cohen’s *d*. * *p* < 0.05.

The results showed more limited sensitivity to SA differences than the eye-tracking indicators. Among the EEG features extracted from the frontal, fronto-central, and parietal regions, only the relative theta power in the fronto-central region during the SA1 perception stage reached statistical significance between the relatively higher-SA and lower-SA groups (*p* = 0.0315), as shown in Figure 6 and Table 5. This finding suggests that fronto-central theta-related activity may be associated with perceptual SA during VDT tasks. In addition, fronto-central relative theta power during the SA2 comprehension and SA3 projection stages showed similar trends, although these differences did not reach the significance threshold (*p* = 0.0599 and *p* = 0.0576, respectively; Appendix A Table A1). Therefore, EEG features provided complementary but more limited evidence of SA-related group differences.

These results indicate that eye-tracking indicators, especially saccade peak velocity and its variability, provided evidence of differences between participants with relatively higher and lower self-reported SA in the VDT tasks. EEG features provided complementary evidence, mainly reflected in fronto-central theta-related activity during the perception stage, with similar but non-significant trends during the comprehension and projection stages. These findings provide preliminary evidence that selected physiological measures may complement subjective SA assessment, while also suggesting that saccade-related eye-tracking features may be particularly useful for detecting SA-related differences in dynamic VDT tasks.

To further examine whether the SA-related findings were consistent when SART was treated as a continuous variable, complementary Spearman correlation analyses were conducted between SART scores and the indicators identified in the group comparisons. As shown in Table A3, all six indicators showed negative correlations with SART scores. Significant correlations were observed for SA2 standard deviation of saccade peak velocity (*ρ* = −0.464, *p* = 0.0099) and SA3 saccade peak velocity (*ρ* = −0.425, *p* = 0.0192). SA1 standard deviation of saccade peak velocity (*ρ* = −0.347, *p* = 0.0600) and SA1 fronto-central relative theta power (*ρ* = −0.350, *p* = 0.0581) showed near-threshold trends. These results indicate that higher self-reported SA tended to be associated with lower saccade velocity or lower saccade-velocity variability, supporting the group-based finding that lower SA was associated with less stable visual scanning behavior.

Exploratory linear regression analyses using continuous SART scores showed directions generally consistent with the Spearman correlation analyses. Among the SA-sensitive indicators in the group-based comparisons, SA1 SD of saccade peak velocity was significantly associated with SART score (standardized β = −0.403, R^2^ = 0.162, *p* = 0.0273) in the exploratory simple linear regression model, as did SA2 SD of saccade peak velocity (standardized β = −0.443, R^2^ = 0.197, *p* = 0.0141) and SA3 saccade peak velocity (standardized β = −0.376, R^2^ = 0.141, *p* = 0.0405). These results suggest that higher saccade velocity variability or higher saccade peak velocity was associated with lower self-reported SA when SART was treated as a continuous variable. Other indicators showed negative but non-significant regression coefficients. Therefore, the continuous-score regression analyses provided supplementary support for the SA-related eye-tracking findings and reduced the dependence of interpretation on dichotomous SART-based grouping.

### 3.2. Associations Between Perceived Mental Workload and SA-Related Outcomes

To examine workload-related differences, participants were categorized into relatively higher-workload and lower-workload groups according to their NASA-TLX scores. This comparison was used as a descriptive group-based analysis and was supplemented by continuous-score correlation and regression analyses. The higher-workload group included 16 participants, and the lower-workload group included 14 participants. Subjective SA, behavioral performance, EEG features, and eye-tracking indicators were then compared between the two groups.

**Figure 7:**
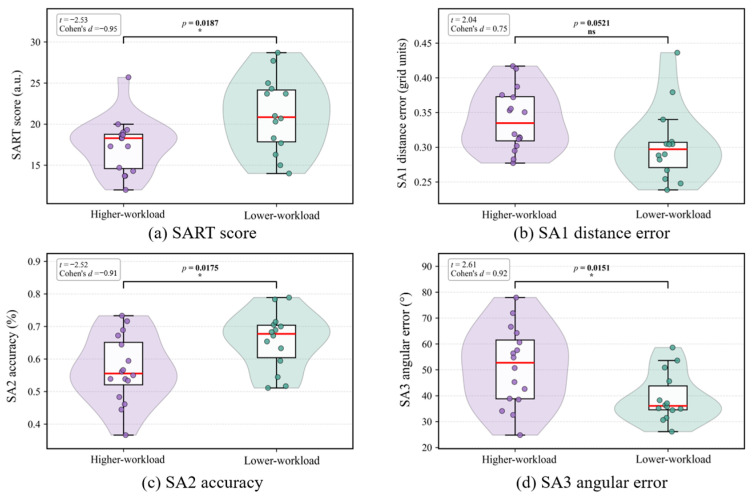
Subjective and behavioral differences between relatively higher-workload and lower-workload groups. (**a**) SART score (a.u.), (**b**) SA1 distance error (grid units), (**c**) SA2 accuracy (%), and (**d**) SA3 angular error (°). Lower values indicate better performance for SA1 distance error and SA3 angular error, whereas higher values indicate better performance for SART score and SA2 accuracy. Statistical annotations indicate *p* value and Cohen’s *d*. * *p* < 0.05; ns, not significant.

The results showed that the higher-workload group reported significantly lower SART scores than the lower-workload group, indicating that participants who perceived greater task demand also reported lower SA. As shown in Table 6 and Figure 7, the higher-workload group had a significantly lower SART score than the lower-workload group (*p* = 0.0187). This result suggests that higher perceived workload was associated with reduced self-reported awareness of the task environment.

Behavioral performance further supported this relationship. In the SA2 comprehension task, the higher-workload group showed significantly lower accuracy than the lower-workload group (*p* = 0.0175), indicating that higher perceived workload was associated with lower comprehension accuracy for task-relevant visual information. In the SA3 projection task, the higher-workload group showed significantly larger angular prediction errors than the lower-workload group (*p* = 0.0151), indicating poorer projection performance among participants reporting higher perceived workload. In the SA1 perception task, the higher-workload group also showed larger distance errors than the lower-workload group, although this difference was marginal and did not reach the conventional significance threshold (*p* = 0.0521).

These findings indicate that perceived workload was associated with poorer SA-related performance, particularly at the comprehension and projection stages. The association was less evident for basic perception but more evident when participants were required to integrate object information and predict future movement. This pattern suggests that higher-level SA processes may be more vulnerable to perceived workload than simple perceptual localization.

**Figure 8:**
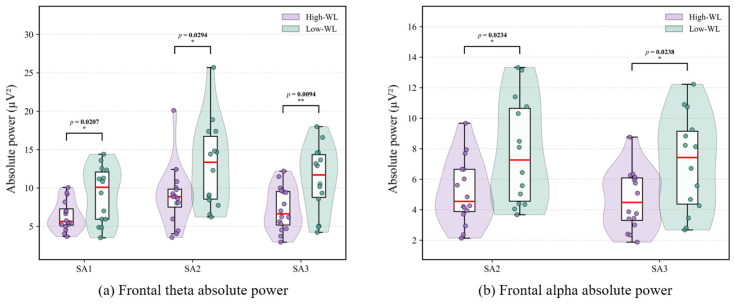
EEG features showing workload-related differences. (**a**) Frontal theta absolute power (${\mu V}^{2}$) during the SA1, SA2, and SA3 stages. (**b**) Frontal alpha absolute power (${\mu V}^{2}$) during the SA2 and SA3 stages. Relatively higher-workload and lower-workload groups are compared within each SA stage. Bars represent mean ± SEM, and annotations indicate *p* values and Cohen’s *d*. * *p* < 0.05; ** *p* < 0.01.

EEG features provided additional physiological evidence for workload-related differences. As shown in Figure 8 and Table 7, frontal theta-related and alpha-related absolute power features differed significantly between the higher-workload and lower-workload groups. Specifically, frontal theta power differed significantly during the SA1 perception stage (*p* = 0.0207), SA2 comprehension stage (*p* = 0.0294), and SA3 projection stage (*p* = 0.0094). Frontal alpha absolute power was also significantly lower in the higher-workload group during the SA2 comprehension stage (*p* = 0.0234) and SA3 projection stage (*p* = 0.0238). These results suggest that perceived workload was accompanied by changes in frontal EEG spectral activity during the VDT task.

In contrast, eye-tracking indicators did not show significant differences between the higher-workload and lower-workload groups in the current analysis. As shown in Table A2, none of the eye-tracking indicators reached statistical significance, with *p* values ranging from 0.2260 to 0.9225. The smallest *p* value was observed for SA3 mean saccade peak velocity (*p* = 0.2260), which still did not approach the conventional significance threshold. Although the saccade-related indicators showed statistically reliable differences in the SA-based comparisons in Section 3.1, they did not show statistically reliable differences between the NASA-TLX-based workload groups. The selected eye-tracking features showed clearer differences in the SA-based comparisons than in the NASA-TLX-based workload comparisons in this dataset.

Complementary Spearman correlation analyses were also conducted using continuous NASA-TLX scores. As shown in Table A4, NASA-TLX scores were significantly negatively correlated with SART scores (*ρ* = −0.435, *p* = 0.0162) and SA2 accuracy (*ρ* = −0.462, *p* = 0.0102), and positively correlated with SA1 distance error (*ρ* = 0.362, *p* = 0.0497) and SA3 angular error (*ρ* = 0.419, *p* = 0.0212). NASA-TLX scores were also significantly negatively correlated with several frontal EEG indicators associated with perceived workload, including SA1 theta absolute power (*ρ* = −0.468, *p* = 0.0091), SA2 theta absolute power (*ρ* = −0.370, *p* = 0.0442), SA3 theta absolute power (*ρ* = −0.524, *p* = 0.0029), SA2 alpha absolute power (*ρ* = −0.386, *p* = 0.0354), and SA3 alpha absolute power (*ρ* = −0.440, *p* = 0.0150). These continuous-score associations were consistent with the high- versus lower-workload group comparisons and suggest that the workload-related findings were not solely dependent on dichotomous grouping.

Exploratory linear regression analyses using continuous NASA-TLX scores further supported the workload-related findings. Significant regression associations with NASA-TLX scores were observed for SART score (standardized β = −0.432, R^2^ = 0.186, *p* = 0.0172), SA1 distance error (standardized β = 0.401, R^2^ = 0.160, *p* = 0.0283), and SA2 accuracy (standardized β = −0.472, R^2^ = 0.223, *p* = 0.0084). Frontal EEG indicators associated with perceived workload also showed significant regression relationships with NASA-TLX scores, including SA1 frontal theta absolute power (standardized β = −0.501, R^2^ = 0.251, *p* = 0.0048), SA3 frontal theta absolute power (standardized β = −0.552, R^2^ = 0.304, *p* = 0.0016), SA2 frontal alpha absolute power (standardized β = −0.495, R^2^ = 0.245, *p* = 0.0055), and SA3 frontal alpha absolute power (standardized β = −0.569, R^2^ = 0.324, *p* = 0.0010). These continuous-score regression results were consistent with the group-based comparisons. Therefore, the workload-related findings were interpreted based on both NASA-TLX-based group comparisons and continuous-score associations, rather than on dichotomous grouping alone.

Overall, relatively higher perceived workload was associated with lower SA in VDT tasks, and this relationship was supported by both group-based comparisons and continuous-score correlation analyses. This association was supported by lower SART scores, lower SA2 comprehension accuracy, larger SA3 projection errors, and significant frontal EEG differences in the higher-workload group. The significant EEG indicators showed large effect sizes, with Cohen’s *d* values ranging from −0.872 to −1.073. In contrast, the selected eye-tracking indicators did not reach statistical significance under NASA-TLX-based perceived workload grouping in this dataset, with *p* values ranging from 0.2260 to 0.9225. Therefore, the workload-related pattern was reflected primarily in subjective, behavioral, and EEG measures, whereas the selected eye-tracking indicators showed no statistically reliable differences under NASA-TLX-based grouping.

### 3.3. Exploratory Association of Incentive Conditions with SA

Exploratory comparisons were conducted to examine whether short-term incentive conditions were associated with differences in overall subjective SA and stage-specific behavioral performance. Participants were divided into positive-incentive, negative-incentive, and no-incentive groups. One-way ANOVA was conducted to compare overall SART scores and behavioral indicators corresponding to the SA1 perception, SA2 comprehension, and SA3 projection stages.

**Figure 9:**
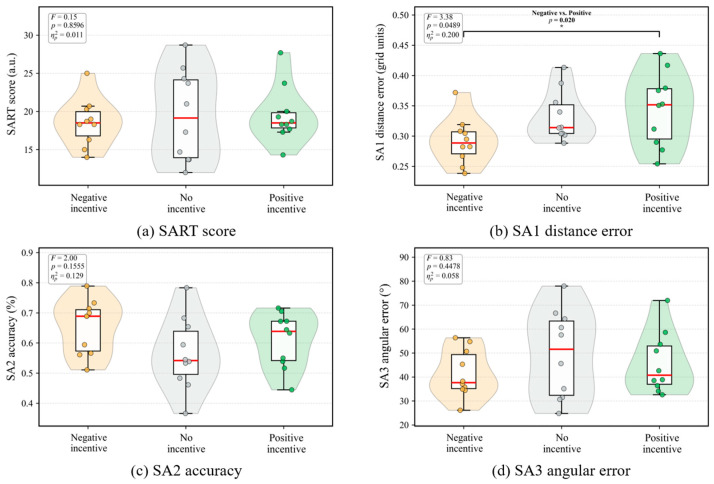
Exploratory comparisons of SA indicators across incentive conditions. (**a**) SART score (a.u.), (**b**) SA1 distance error (grid units), (**c**) SA2 accuracy (%), and (**d**) SA3 angular error (°) under negative-incentive, no-incentive, and positive-incentive conditions. Bars represent mean ± SEM. Statistical annotations indicate *F*, *p*, and $\eta_{p}^{2}$. The bracket in panel (**b**) indicates the post hoc comparison between the negative- and positive-incentive groups. * *p* < 0.05.

As shown in Table 8 and Figure 9, the mean SART scores were 18.56 ± 3.13 in the negative-incentive group, 19.48 ± 5.94 in the no-incentive group, and 19.53 ± 3.71 in the positive-incentive group. No statistically reliable difference was detected among the three groups, *F*(2, 27) = 0.152, *p* = 0.860, $\eta_{p}^{2}$ = 0.011. Thus, no statistically reliable between-group difference was detected in overall self-reported SA under the present short-term incentive conditions.

For the SA1 perception task, an exploratory stage-specific difference was observed in distance error. The mean distance errors were 0.29 ± 0.04 in the negative-incentive group, 0.33 ± 0.04 in the no-incentive group, and 0.34 ± 0.06 in the positive-incentive group. The omnibus ANOVA indicated a difference among the three groups, *F* = 3.382, *p* = 0.049, $\eta_{p}^{2}$ = 0.200. As shown in Table 9, post hoc comparisons showed that the negative-incentive group had a lower SA1 distance error than the positive-incentive group, with a mean difference of −0.053 (*p* = 0.020). The difference between the negative-incentive and no-incentive groups did not reach statistical significance (*p* = 0.066), and no statistically reliable difference was detected between the no-incentive and positive-incentive groups (*p* = 0.575). Given the exploratory design and limited subgroup size, this finding was interpreted as a limited stage-specific pattern.

No statistically reliable differences were detected for the SA2 comprehension or SA3 projection indicators. For SA2 comprehension, the mean accuracy values were 0.65 ± 0.09, 0.56 ± 0.12, and 0.61 ± 0.09 for the negative-incentive, no-incentive, and positive-incentive groups, respectively; the result was not significant, *F*(2, 27) = 1.995, *p* = 0.156, $\eta_{p}^{2}$ = 0.129. For SA3 projection, the angular errors were 41.38 ± 9.94, 49.47 ± 18.33, and 45.83 ± 12.67, respectively, *F*(2, 27) = 0.828, *p* = 0.448, $\eta_{p}^{2}$ = 0.058.

Overall, the exploratory analyses identified one limited and stage-specific difference in SA1 distance error, while no reliable differences were detected in overall SART score, SA2 accuracy, or SA3 angular error. Given the limited statistical power for small-to-medium incentive-related effects, these findings provide insufficient evidence that the present short-term instruction-based manipulation produced reliable regulation of SA.

### 3.4. Exploratory Association of Incentive Conditions with Perceived Mental Workload

An exploratory comparison was conducted to examine whether short-term incentive conditions were associated with differences in perceived mental workload. NASA-TLX scores were compared among the negative-incentive, no-incentive, and positive-incentive groups using one-way ANOVA. As noted above, the subgroup size of 10 participants per condition limited the statistical power for detecting small-to-medium incentive-related differences.

**Figure 10:**
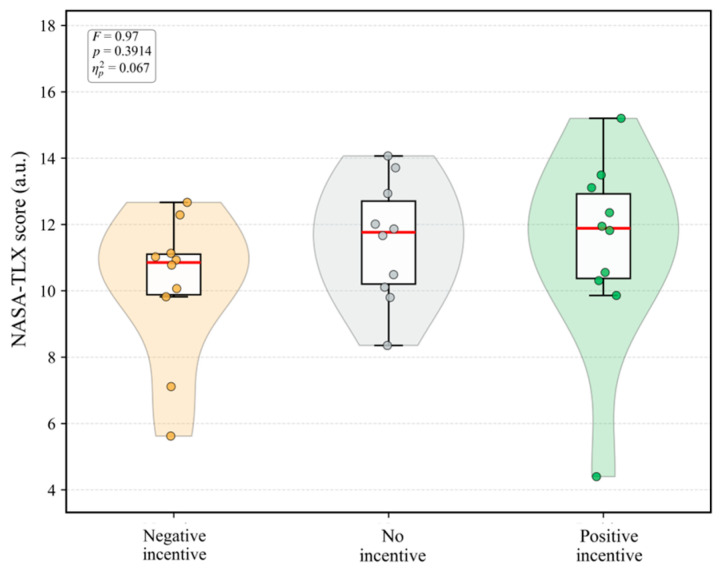
Exploratory comparison of perceived mental workload across incentive conditions. NASA-TLX scores (a.u.) are compared among negative-incentive, no-incentive, and positive-incentive conditions. Bars represent mean ± SEM, and statistical annotations indicate *F*, *p*, and $\eta_{p}^{2}$.

As shown in Figure 10 and Table 10, the mean NASA-TLX score was 10.144 ± 2.198 in the negative-incentive group, 11.501 ± 1.816 in the no-incentive group, and 11.305 ± 2.912 in the positive-incentive group. No statistically reliable difference was detected among the three groups, *F*(2, 27) = 0.971, *p* = 0.391, $\eta_{p}^{2}$ = 0.067. Therefore, the present data provide insufficient evidence of an association between the short-term incentive conditions and post-task perceived workload.

Together with Section 3.3, these findings indicate that the observed incentive-related pattern was limited to SA1 distance error, with no reliable differences in overall SART score, SA2 accuracy, SA3 angular error, or NASA-TLX score. Within the statistical boundaries of the present study, the results provide insufficient evidence for broader cognitive-state regulation but should not be interpreted as evidence that incentive strategies are generally ineffective.

## 4. Discussion

### 4.1. Multimodal Indicators for SA Assessment

This study examined whether EEG and eye-tracking indicators could support the assessment of SA in VDT tasks. The results showed that saccade-related eye-tracking indicators, especially mean saccade peak velocity and the standard deviation of saccade peak velocity, showed differences between relatively higher-SA and lower-SA groups. Participants in the lower-SA group showed higher saccade peak velocity and greater variability, suggesting less stable scanning behavior during the dynamic VDT task. This finding is consistent with previous eye-tracking studies showing that gaze, saccade, and pupil metrics can reflect visual attention allocation, scanning strategy, and cognitive workload in monitoring-related tasks [46,50]. In the SATEST-based VDT task, participants were required to track moving objects, retain spatial information, identify object-related cues, and predict future movement. These processes depend on effective visual search and attention allocation. Therefore, higher saccade velocity and variability may indicate a less organized scanning strategy or greater difficulty in maintaining attention to task-relevant information.

The EEG results provided complementary but limited evidence. Only fronto-central relative theta power during the SA1 perception stage reached statistical significance between relatively higher-SA and lower-SA groups, while similar theta-related effects during SA2 and SA3 did not reach significance. This pattern suggests that fronto-central relative theta activity may be related to perceptual-stage attentional engagement, but it should not be interpreted as a general EEG marker of all SA levels. In the present dataset, EEG features were less sensitive than eye-tracking indicators for distinguishing SA levels, indicating that the two modalities contributed unequally to SA assessment. Overall, the results provide preliminary support for the use of multimodal physiological assessment for SA assessment, consistent with previous studies showing that EEG and eye-tracking fusion can provide complementary information for cognitive workload or attention-state monitoring [37,67].

### 4.2. Associations Between Perceived Workload and SA-Related Outcomes

The results showed that higher perceived workload was associated with lower SA. Participants in the higher-workload group reported significantly lower SART scores, lower SA2 comprehension accuracy, and larger SA3 projection errors. SA1 perception error was also higher in the higher-workload group, although this difference was marginal. Therefore, the workload-related behavioral findings were interpreted as strongest for SA2 comprehension and SA3 projection, whereas the SA1 perception result was treated as a marginal trend. These findings suggest that perceived workload may be more closely associated with higher-level SA processes, especially comprehension and projection, than with basic perceptual localization. This pattern is compatible with the cognitive demands of the three SA levels. SA1 perception mainly requires the detection and retention of object locations, whereas SA2 and SA3 require participants to integrate object information and anticipate future movement. When perceived workload increases, fewer cognitive resources may remain available for information integration and prediction, which is consistent with multiple resource theory and previous neuroergonomic accounts of workload-related performance degradation [13,33]. However, because workload was assessed using post-task NASA-TLX scores rather than experimentally manipulated task difficulty, this interpretation should be regarded as an association rather than a direct causal effect.

EEG features provided additional but preliminary evidence for workload-related differences. Several frontal theta-related and alpha-related absolute power features differed between relatively higher-workload and lower-workload groups. This is consistent with previous evidence that frontal theta and alpha-band activity are sensitive to cognitive workload, executive control, and attentional regulation [41]. However, the direction of these effects should be interpreted cautiously. Many workload studies have reported increased frontal theta activity under higher objectively manipulated task demands. In the present study, however, workload was defined according to post-task NASA-TLX scores rather than experimentally manipulated task difficulty, time pressure, or information load. Therefore, the lower frontal theta and alpha absolute power observed in the relatively higher-workload group should not be interpreted as a universal neural signature of increased objective task demand.

A possible explanation is that NASA-TLX-based perceived workload may reflect not only task demand but also fatigue, reduced cognitive reserve, lower task engagement, frustration, and individual differences in effort appraisal. Under such conditions, reduced frontal theta and alpha absolute power may indicate a less efficient or less engaged cognitive state rather than the classic frontal theta enhancement observed during active compensatory control. Therefore, the present EEG findings should be regarded as preliminary evidence that perceived workload was associated with altered frontal spectral activity.

The absence of statistically reliable eye-tracking differences between the NASA-TLX-based workload groups should be interpreted cautiously and should not be taken to indicate that eye movements are generally unrelated to cognitive workload. In the present study, the selected eye-tracking indicators were extracted separately from the SA1, SA2, and SA3 task windows and therefore reflected stage-specific visual behavior during perception, comprehension, and projection. By contrast, NASA-TLX was administered after task completion and provided a retrospective and global assessment of perceived workload. This difference in temporal resolution and construct measurement may have reduced the correspondence between stage-specific eye movements and post-task workload grouping. The selected saccade-related indicators may also have been more closely related to the visual scanning requirements of the SATEST task than to global perceived workload [68]. Mean saccade peak velocity and its variability characterize aspects of gaze-shift dynamics and scanning stability, which may be particularly relevant when participants must continuously locate, identify, and predict the movement of visual objects. This may explain why these indicators showed clearer differences in the SA-based comparisons than in the NASA-TLX-based workload comparisons. However, this pattern does not establish that the selected indicators are uniquely specific to SA.

Alternative explanations should also be considered. First, the modest sample size and the relatively higher-workload and lower-workload group sizes limited the statistical power for detecting small-to-medium eye-tracking differences; therefore, smaller workload-related associations may have remained undetected. Second, workload was not experimentally manipulated through task difficulty, information density, time pressure, or dual-task demand. The NASA-TLX grouping therefore reflected individual differences in post-task perceived workload rather than controlled differences in objective task demand. Third, other eye-tracking features, including fixation duration and count, blink behavior, task-evoked pupil changes, gaze transitions, and scanpath entropy, may be more sensitive to workload under some experimental conditions [46]. Accordingly, the present findings should be interpreted as task-specific, feature-specific, and measurement-specific. They suggest that the selected saccade-related indicators were more informative for SA-related visual scanning differences than for NASA-TLX-based perceived workload grouping in this dataset, rather than demonstrating that eye-tracking metrics are generally insensitive to workload.

### 4.3. Exploratory and Stage-Specific Incentive-Related Patterns

The exploratory incentive analysis identified one limited and stage-specific pattern, with a difference in SA1 distance error but no statistically reliable differences in overall SART score, SA2 accuracy, SA3 angular error, or NASA-TLX score. The negative-incentive group showed a lower SA1 distance error than the positive-incentive group. However, given the small subgroup size, limited statistical power, and absence of a manipulation check, this isolated finding should be interpreted as a preliminary stage-specific pattern rather than as robust evidence of an incentive effect.

One possible explanation is that the negative-incentive instruction may have heightened vigilance or error-avoidance motivation during the relatively simple perceptual localization task. This interpretation is consistent with the view that moderate arousal or pressure can facilitate simple task performance, whereas reward and punishment may induce different cognitive control strategies [69,70]. However, motivation, arousal, and perceived task importance were not measured directly, and this interpretation therefore remains speculative. No corresponding reliable differences were observed in comprehension, projection, overall self-reported SA, or perceived workload. Accordingly, the present results provide insufficient evidence that the short-term incentive manipulation produced broad or reliable changes in cognitive state.

These findings should not be interpreted as evidence that incentives are irrelevant to VDT performance. Rather, incentive-related outcomes may depend on task duration, incentive intensity, feedback timing, individual motivation, and whether the task primarily requires speed, accuracy, working memory, or higher-level integration. Previous meta-analytic evidence suggests that extrinsic rewards can affect intrinsic motivation and performance in complex ways, rather than producing uniformly beneficial effects [71,72]. Recent cognitive studies further indicate that reward and punishment can influence cognitive control and visual working memory [53,70]. In the present study, reward and punishment instructions functioned as exploratory contextual factors and showed only a limited pattern. This is consistent with the possibility that short-term instruction-based incentives alone may be insufficient to produce stable changes in operators’ cognitive states under the current experimental conditions [73]. The observed pattern therefore defines the limited scope of the present analysis and requires confirmation in a larger and adequately powered experiment.

### 4.4. Implications, Limitations, and Future Work

The findings provide preliminary implications for technology-assisted cognitive-state assessment in VDT tasks. Traditional subjective scales and behavioral indicators remain essential, but they are primarily retrospective or outcome-oriented. EEG and eye-tracking may provide complementary process-level information about neural activity and visual behavior during task execution, thereby informing the future development of operator-state assessment methods in human–machine systems [49,50]. Saccade peak velocity and its variability emerged as candidate indicators of SA-related visual scanning differences, whereas EEG features provided preliminary information about workload-related spectral changes. However, these measures should not be regarded as validated inputs for real-time cognitive-state monitoring or workload regulation. The unequal sensitivity observed across modalities also supports the use of multimodal assessment, because no single physiological measure is likely to characterize SA and perceived workload consistently across all task contexts [19,74]. Future research may examine whether combining subjective, behavioral, EEG, and eye-tracking information improves operator-state assessment across different VDT tasks. Workload-support approaches, including interface simplification, alarm prioritization, task redistribution, and appropriately timed recovery recommendations, could also be evaluated in future adaptive-automation studies [10,75].

Several limitations are inherent to the present experimental design and cannot be remedied through additional statistical analysis. First, the overall sample size was modest, and the three incentive-condition subgroups included only 10 participants each, leaving the incentive analyses underpowered for small-to-medium effects. Second, the incentive manipulation consisted of brief reward or punishment instructions and did not include repeated reinforcement, performance-contingent feedback, or an independent manipulation check. Third, workload was assessed using a retrospective post-task NASA-TLX score rather than an objectively manipulated workload factor. Consequently, the observed stage-specific difference in SA1 distance error and the remaining non-significant incentive findings cannot establish either the effectiveness or ineffectiveness of incentive-based cognitive-state regulation. Similarly, associations between NASA-TLX scores and physiological or behavioral outcomes should not be interpreted as direct causal effects of objectively increased workload. These design characteristics define the boundaries of inference for the present study. Accordingly, the findings should be interpreted as preliminary and task-specific associations within a controlled VDT simulation, rather than as definitive validation of real-time cognitive-state monitoring, workload regulation, or an effective incentive-based intervention.

Beyond these core boundaries of inference, the overall sample size also limits statistical precision and model development. Although a total sample of 30 participants is within the range commonly reported in EEG-based and eye-tracking-based neuroergonomic studies, it is insufficient for robustly estimating small effects, complex interactions, stable subgroup differences, or generalizable multivariable prediction models. A recent systematic review of concurrent EEG and eye-tracking studies reported that, across 47 included studies, the number of participants ranged from 8 to 150. After excluding one upper outlier with 150 participants, the revised range was 8 to 61 participants, with a mean of 22.83 ± 14.17 [30,38]. Thus, the present sample is comparable to those used in previous studies, but comparability does not eliminate the limitations associated with statistical power and estimate uncertainty. Larger samples would provide more stable estimates of effect sizes and correlations, narrower confidence intervals, and greater power to detect small-to-medium associations.

The participant population and experimental task also limit generalizability. Participants were healthy young adults rather than professional VDT operators. Professional experience may affect visual scanning strategies, task prioritization, workload appraisal, and the neural resources used to maintain SA. In addition, although the SATEST task provides a controlled and repeatable paradigm for examining perception, comprehension, and projection in a VDT-like setting, it cannot reproduce the full complexity of operational monitoring and control environments. Real VDT work may involve heterogeneous information sources, multiple alarm types, time-critical decisions, interruptions, organizational procedures, and meaningful operational consequences. Therefore, the identified physiological measures should be treated as candidate indicators requiring validation with professional operators in high-fidelity simulations and field-based monitoring scenarios.

The measurement and analysis of perceived workload introduce further limitations. NASA-TLX was administered after task completion and reflected participants’ retrospective and global appraisal of workload. It may therefore have captured not only task demand, but also fatigue, effort, frustration, engagement, and individual differences in self-evaluation. It may also have failed to represent stage-specific fluctuations occurring during the SA1, SA2, and SA3 task periods. For this reason, the workload-related EEG and eye-tracking findings should not be interpreted as universal markers of objectively increased task demand. Future studies should experimentally manipulate workload through task difficulty, information density, time pressure, dual-task demand, or event frequency, while simultaneously collecting stage-level or trial-level workload measures. In addition, SART and NASA-TLX scores were also dichotomized into relatively higher and lower groups for visualization and group-based descriptive comparisons. Although this approach facilitated the presentation of physiological differences between participants with different levels of self-reported SA and perceived workload, dichotomization may reduce statistical power, discard information, and introduce arbitrary group boundaries. To mitigate this limitation, the group-based analyses were supplemented with Spearman correlations and exploratory linear regression models using the original continuous SART and NASA-TLX scores. These continuous-score analyses were generally consistent with the principal group-based findings, particularly for NASA-TLX-related associations. Nevertheless, future studies with larger samples should use multivariable generalized linear models, mixed-effects models, and cross-validated predictive analyses to evaluate continuous and repeated-measures relationships more rigorously.

Response time was not included in the final statistical analysis. Although response speed may provide useful information about task efficiency and speed-accuracy trade-offs, complete and reliable trial-level response-time records were not available in the exported dataset used for this study. Therefore, the behavioral analysis focused on SA1 distance error, SA2 accuracy, and SA3 angular error, which directly corresponded to the three SA levels. Future studies should retain and analyze response-time data to better distinguish task accuracy, processing efficiency, and speed-accuracy trade-offs.

Future research should address the incentive-design limitations identified above by adopting stronger and verifiable manipulations, including trial-by-trial performance feedback, performance-contingent rewards or penalties, repeated reinforcement schedules, longer manipulation periods, and manipulation checks assessing motivation, stress, arousal, and perceived task importance. A broader feature set, including EEG connectivity, fixation transitions, scanpath entropy, blink behavior, task-evoked pupil responses, and pupil change rate, may provide additional information in future validation studies [46,76]. Overall, future validation should involve larger and more diverse samples, professional VDT operators, objectively controlled workload conditions, adequately powered incentive designs, and high-fidelity or field-based tasks. Such validation is necessary before the identified measures can be considered sufficiently reliable for practical operator-state assessment.

## 5. Conclusions

This study applied a multimodal physiological assessment framework for examining SA and perceived mental workload in a SATEST-based VDT simulation task by integrating EEG, eye-tracking, subjective scales, and behavioral performance measures. The results indicated that saccade-related eye-tracking indicators, particularly saccade peak velocity and the standard deviation of saccade peak velocity, differed between participants with relatively higher and lower self-reported SA. Fronto-central relative theta power also showed an SA-related difference during the SA1 stage, although the EEG evidence was more limited. These findings suggest that the identified eye-tracking and EEG measures may serve as candidate physiological indicators of SA-related visual scanning and neural activity, but they require further validation.

The findings also suggested that higher perceived workload was associated with lower subjective SA, poorer SA2 comprehension performance, larger SA3 projection errors, and altered frontal EEG spectral activity. These results indicate that perceived workload may be related to reduced performance in higher-level SA processes, especially comprehension and projection. However, because workload was assessed using retrospective post-task NASA-TLX scores rather than experimentally manipulated task difficulty, these findings should be interpreted as associations rather than direct causal effects. The selected eye-tracking indicators showed no statistically reliable differences under NASA-TLX-based workload grouping. Therefore, this result should not be interpreted as evidence that eye-tracking metrics are generally unrelated to workload, but rather as a task- and feature-specific finding under the present SATEST-based VDT task. The incentive-related analysis was exploratory, underpowered for small-to-medium effects, and was not designed to validate an incentive-based cognitive-state regulation strategy. It identified one limited and stage-specific difference in SA1 distance error, while no statistically reliable differences were detected in overall SART score, SA2 accuracy, SA3 angular error, or NASA-TLX score. Therefore, the present findings provide insufficient evidence that the short-term instruction-based incentive manipulation produced reliable cognitive-state regulation. These preliminary findings do not establish an effective incentive-based intervention.

Overall, this study provides preliminary evidence that EEG and eye-tracking can complement subjective and behavioral measures in VDT cognitive-state assessment. The physiological measures identified in this study should be regarded as candidate indicators requiring further validation, rather than as validated inputs for real-time cognitive-state monitoring, workload regulation, or adaptive intervention. Future studies should also use larger samples, recruit professional operators, introduce controlled workload manipulations, adopt stronger incentive designs, and include broader eye-tracking features, such as fixation duration, blink behavior, gaze transition, scanpath entropy, and pupil dynamics, to evaluate the candidate indicators in high-fidelity simulations and field-based VDT settings.

## Figures and Tables

**Table 1:** Participant and apparatus information.

Item	Description
Participants	30 adults
Age	21 ± 3 years
EEG system	Neuroscan Grael EEG 2
EEG channels	32 channels
EEG sampling rate	1024 Hz
Electrode placement	International 10–20 system
Eye tracker	Tobii Glasses 2
Eye-tracking sampling rate	50 Hz
Scene video	1920 × 1080, 25 fps
Task software	PEBL version 2.1 (SATEST)
Subjective measures	SART, NASA-TLX

**Table 2:** Behavioral indicators for SA measurement.

SA Level	Task	Behavioral Indicator	Interpretation
SA1—Perception	Click final object locations	Distance error	Higher distance error indicates poorer perception
SA2—Comprehension	Identify object type at highlighted positions	Accuracy	Lower accuracy indicates poorer comprehension
SA3—Projection	Predict future movement direction	Angular error	Higher angular error indicates poorer projection

**Table 3:** Summary of extracted multimodal features.

Modality	Feature Category	Extracted Indicators	Analysis Stage
Subjective scale	SA	SART score	Post-task
	Workload	NASA-TLX score	
Behavioral performance	SA1 perception	Distance error	SA1
	SA2 comprehension	Accuracy	SA2
	SA3 projection	Angular error	SA3
EEG	Absolute power	δ, θ, α, β absolute power	SA1, SA2, SA3
	Relative power	δ, θ, α, β relative power	
	Ratio indices	θ/β; α/β; (θ + α)/β; (θ + α)/(β + α)	
Eye-tracking	Saccade features	Mean saccade peak velocity; SD of saccade peak velocity; saccade amplitude; saccade frequency	
	Pupil feature	Mean pupil diameter	

**Table 4:** Eye-tracking indicators differing between relatively higher-SA and lower-SA groups.

SA Stage	Eye-Tracking Indicator	Higher-SA Group	Lower-SA Group	*t*	*p*	Cohen’s *d*
SA1 Perception	SD of saccade peak velocity	77.003 ± 10.207	102.861 ± 32.085	−3.186	0.0043	−1.002
SA2 Comprehension	SD of saccade peak velocity	100.456 ± 16.345	118.578 ± 19.599	−2.744	0.0107	−0.985
SA3 Projection	Mean saccade peak velocity	232.084 ± 25.520	258.382 ± 33.919	−2.419	0.0224	−0.851
SA1 Perception	Mean saccade peak velocity	190.691 ± 15.450	205.598 ± 18.648	−2.381	0.0247	−0.854
SA3 Projection	SD of saccade peak velocity	97.338 ± 16.522	122.605 ± 46.517	−2.113	0.0457	−0.670

Note: Values are presented as mean ± SD. The higher-SA group included 12 participants, and the lower-SA group included 18 participants. Negative *t* and Cohen’s *d* values indicate lower values in the higher-SA group than in the lower-SA group.

**Table 5:** EEG indicator showing a significant SA-related difference.

SA Stage	Brain Region	EEG Indicator	Higher-SA Group	Lower-SA Group	*t*	*p*	Cohen’s *d*
SA1 Perception	Fronto-central region	Relative theta power	0.092 ± 0.016	0.112 ± 0.032	−2.273	0.0315	−0.746

Note: Values are presented as mean ± SD. The higher-SA group included 12 participants, and the lower-SA group included 18 participants. Negative *t* and Cohen’s *d* values indicate lower values in the higher-SA group than in the lower-SA group.

**Table 6:** Subjective and behavioral differences between higher-workload and lower-workload groups.

Indicator	Higher-Workload Group	Lower-Workload Group	*t*	*p*	Cohen’s *d*
SART score	17.456 ± 3.277	21.171 ± 4.567	−2.528	0.0187	−0.946
SA1 distance error	0.340 ± 0.044	0.303 ± 0.053	2.037	0.0521	0.755
SA2 accuracy	0.568 ± 0.103	0.656 ± 0.088	−2.525	0.0175	−0.914
SA3 angular error	51.108 ± 15.225	39.217 ± 9.408	2.607	0.0151	0.925

Note: Values are presented as mean ± SD. The higher-workload group included 16 participants, and the lower-workload group included 14 participants. Positive *t* and Cohen’s *d* values indicate higher values in the higher-workload group, whereas negative values indicate lower values in the higher-workload group.

**Table 7:** EEG features showing significant workload-related differences.

SA Stage	Brain Region	EEG Indicator	Higher-Workload Group	Lower-Workload Group	*t*	*p*	Cohen’s *d*
SA3 Projection	Frontal region	Theta absolute power	7.287 ± 2.819	11.174 ± 4.372	−2.849	0.0094	−1.073
SA1 Perception			6.372 ± 1.950	9.127 ± 3.662	−2.520	0.0207	−0.959
SA2 Comprehension			8.886 ± 3.885	13.043 ± 5.620	−2.324	0.0294	−0.872
SA3 Projection		Alpha absolute power	4.656 ± 1.913	7.035 ± 3.181	−2.440	0.0238	−0.922
SA2 Comprehension			5.177 ± 2.132	7.792 ± 3.471	−2.444	0.0234	−0.923

Note: Values are presented as mean ± SD. The higher-workload group included 16 participants, and the lower-workload group included 14 participants. Negative *t* and Cohen’s *d* values indicate lower EEG feature values in the higher-workload group.

**Table 8:** Exploratory comparison of subjective and SA indicators across incentive conditions.

Indicator	Negative Incentive	No Incentive	Positive Incentive	*F*	*p*	$\eta_{p}^{2}$
SART score	18.56 ± 3.13	19.48 ± 5.94	19.53 ± 3.71	0.152	0.860	0.011
SA1 distance error	0.29 ± 0.04	0.33 ± 0.04	0.34 ± 0.06	3.382	0.049	0.200
SA2 accuracy	0.65 ± 0.09	0.56 ± 0.12	0.61 ± 0.09	1.995	0.156	0.129
SA3 angular error	41.38 ± 9.94	49.47 ± 18.33	45.83 ± 12.67	0.828	0.448	0.058

Note: Values are reported as mean ± SD. $\eta_{p}^{2}$ denotes partial eta squared for the one-way ANOVA. Each incentive group included 10 participants. The analyses were exploratory and underpowered for small-to-medium incentive-related effects.

**Table 9:** Post hoc comparisons for SA1 distance error among incentive conditions.

Comparison	Mean Difference	*p*
Negative incentive vs. No incentive	−0.041	0.066
Negative incentive vs. Positive incentive	−0.053	0.020
No incentive vs. Positive incentive	−0.012	0.575

Note: The mean difference was calculated as the former group minus the latter group. A negative value indicates lower SA1 distance error in the former group.

**Table 10:** Exploratory comparison of perceived mental workload across incentive conditions.

Indicator	Negative Incentive	No Incentive	Positive Incentive	*F*	*p*	$\eta_{p}^{2}$
NASA-TLX score	10.144 ± 2.198	11.501 ± 1.816	11.305 ± 2.912	0.971	0.391	0.067

Note: Values are presented as mean ± SD. Each incentive group included 10 participants. No post hoc comparisons were conducted because the ANOVA result was not statistically significant.

## Data Availability

The data presented in this study are available on request from the corresponding author. The data are not publicly available due to privacy.

## Abbreviations

The following abbreviations are used in this manuscript:

VDT	Visual display terminal
ANOVA	Analysis of variance
EEG	Electroencephalography
ICA	Independent component analysis
NASA-TLX	NASA Task Load Index
PEBL	Psychology Experiment Building Language
PSD	Power spectral density
SA	Situation awareness
SAGAT	Situation Awareness Global Assessment Technique
SART	Situation Awareness Rating Technique
SATEST	Situation Awareness Test
SD	Standard deviation
SPAM	Situation Present Assessment Method
$\eta_{p}^{2}$	Partial eta squared

## Appendix A. Supplementary EEG and Eye-Tracking Comparisons

This appendix provides supplementary statistical results for EEG and eye-tracking indicators that were not fully presented in the main text. Table A1 lists the top-ranked EEG indicators for distinguishing the relatively higher-SA and lower-SA groups. Table A2 presents the complete comparison of eye-tracking indicators between relatively higher-workload and lower-workload groups. In addition, Table A3 and Table A4 provide complementary Spearman correlation analyses using continuous SART and NASA-TLX scores. Table A5 further reports exploratory simple linear regression analyses using continuous SART and NASA-TLX scores as dependent variables. These analyses were included to reduce the potential information loss caused by dichotomizing subjective scores and to examine whether the group-based findings were supported by continuous-score associations.

**Table A1:** EEG indicators differing between relatively higher- and lower-SA groups.

Rank	Indicator	Higher-SA Group	Lower-SA Group	*t*	*p*	Cohen’s *d*	Interpretation
1	Fronto-central relative θ power, SA1	0.092 ± 0.016	0.112 ± 0.032	−2.273	0.0315	−0.746	Significant
2	Fronto-central relative θ power, SA3	0.096 ± 0.018	0.112 ± 0.027	−1.980	0.0576	−0.680	Trend-level only
3	Fronto-central relative θ power, SA2	0.094 ± 0.015	0.109 ± 0.027	−1.963	0.0599	−0.657	Trend-level only
4	Frontal β absolute power, SA3	17.893 ± 8.529	13.190 ± 6.205	1.642	0.1174	0.652	Not significant
5	Parietal relative θ power, SA1	0.089 ± 0.014	0.099 ± 0.023	−1.468	0.1532	−0.499	Not significant

Note: Values are presented as mean ± SD. The higher-SA group included 12 participants, and the lower-SA group included 18 participants. Only fronto-central relative θ power during SA1 reached statistical significance.

**Table A2:** Eye-tracking indicators between relatively higher- and lower-workload groups.

Indicator	Higher-Workload Group	Lower-Workload Group	*t*	*p*	Cohen’s *d*
SA3 mean saccade peak velocity	254.630 ± 37.991	240.129 ± 25.542	1.240	0.2260	0.442
SA3 saccade amplitude	8.648 ± 1.270	8.131 ± 1.320	1.089	0.2859	0.399
SA3 SD of saccade peak velocity	118.778 ± 50.701	105.322 ± 18.550	0.989	0.3350	0.343
SA2 SD of saccade peak velocity	114.611 ± 21.154	107.579 ± 19.119	0.956	0.3471	0.348
SA2 pupil diameter	3.533 ± 0.387	3.713 ± 0.654	−0.904	0.3767	−0.342
SA3 pupil diameter	3.483 ± 0.382	3.635 ± 0.661	−0.757	0.4578	−0.287
SA3 saccade frequency	0.955 ± 0.374	0.847 ± 0.429	0.729	0.4723	0.269
SA1 saccade frequency	0.938 ± 0.330	0.843 ± 0.424	0.678	0.5043	0.252
SA1 pupil diameter	3.505 ± 0.412	3.648 ± 0.690	−0.676	0.5063	−0.256
SA1 SD of saccade peak velocity	95.766 ± 31.071	88.806 ± 25.747	0.671	0.5080	0.242
SA2 saccade frequency	1.440 ± 0.446	1.325 ± 0.541	0.631	0.5334	0.234
SA1 saccade amplitude	6.023 ± 0.612	6.233 ± 1.216	−0.584	0.5663	−0.223
SA2 saccade amplitude	8.231 ± 0.799	8.374 ± 1.023	−0.421	0.6772	−0.157
SA1 mean saccade peak velocity	198.487 ± 19.025	200.947 ± 18.939	−0.354	0.7258	−0.130
SA2 mean saccade peak velocity	249.675 ± 19.052	250.416 ± 21.909	−0.098	0.9225	−0.036

Note: Values are presented as mean ± SD. The higher-workload group included 16 participants, and the lower-workload group included 14 participants. None of the eye-tracking indicators reached statistical significance, supporting the interpretation that eye-tracking features showed limited sensitivity to NASA-TLX-based workload grouping in this study.

**Table A3:** Spearman correlations between SART scores and SA-sensitive physiological indicators.

Indicator	N	Spearman’s *ρ*	*p*	Direction
SA1 saccade peak velocity	30	−0.244	0.1947	Negative
SA1 SD of saccade peak velocity	30	−0.347	0.0600	Negative
SA2 SD of saccade peak velocity	30	−0.464	0.0099	Negative
SA3 saccade peak velocity	30	−0.425	0.0192	Negative
SA3 SD of saccade peak velocity	30	−0.247	0.1876	Negative
SA1 fronto-central relative theta power	30	−0.350	0.0581	Negative

Note: Spearman’s *ρ* was calculated using continuous SART scores and participant-level physiological indicators. Negative correlations indicate that higher SART scores were associated with lower values of the corresponding physiological indicator.

**Table A4:** Spearman correlations between continuous NASA-TLX scores and workload-related indicators.

Indicator	N	Spearman’s *ρ*	*p*	Direction
SART score	30	−0.435	0.0162	Negative
SA1 distance error	30	0.362	0.0497	Positive
SA2 accuracy	30	−0.462	0.0102	Negative
SA3 angular error	30	0.419	0.0212	Positive
SA1 frontal theta absolute power	30	−0.468	0.0091	Negative
SA2 frontal theta absolute power	30	−0.370	0.0442	Negative
SA3 frontal theta absolute power	30	−0.524	0.0029	Negative
SA2 frontal alpha absolute power	30	−0.386	0.0354	Negative
SA3 frontal alpha absolute power	30	−0.440	0.0150	Negative

Note: Spearman’s *ρ* was calculated using continuous NASA-TLX scores and participant-level subjective, behavioral, and EEG indicators. Positive correlations indicate that higher NASA-TLX scores were associated with higher indicator values, whereas negative correlations indicate that higher NASA-TLX scores were associated with lower indicator values.

**Table A5:** Exploratory linear regression analyses using continuous SART and NASA-TLX scores.

Dependent Variable	Predictor	Standardized β	R^2^	*p*
SART score	SA1 saccade peak velocity	−0.285	0.081	0.1275
	SA1 SD of saccade peak velocity	−0.403	0.162	0.0273
	SA2 SD of saccade peak velocity	−0.443	0.197	0.0141
	SA3 saccade peak velocity	−0.376	0.141	0.0405
	SA3 SD of saccade peak velocity	−0.293	0.086	0.1161
	SA1 fronto-central relative theta power	−0.219	0.048	0.2448
NASA-TLX score	SART score	−0.432	0.186	0.0172
	SA1 distance error	0.401	0.160	0.0283
	SA2 accuracy	−0.472	0.223	0.0084
	SA3 angular error	0.346	0.119	0.0615
	SA1 frontal theta absolute power	−0.501	0.251	0.0048
	SA2 frontal theta absolute power	−0.314	0.099	0.0910
	SA3 frontal theta absolute power	−0.552	0.304	0.0016
	SA2 frontal alpha absolute power	−0.495	0.245	0.0055
	SA3 frontal alpha absolute power	−0.569	0.324	0.0010

Note: Separate simple linear regression models were conducted because of the limited sample size. Standardized β indicates the standardized regression coefficient. SART and NASA-TLX scores were treated as continuous variables.
